# Identification of new stable resistant sources and assessing agro-morphological performance of sponge gourd germplasm against *Tomato Leaf curl New Delhi Virus* incidence

**DOI:** 10.3389/fpls.2024.1373352

**Published:** 2024-04-24

**Authors:** Jogendra Singh, Anilabha Das Munshi, Deepak Singh, Bharat Raj Meena, Awani Kumar Singh, Arvind Nagar, Yvonne Angel Lyngdoh, Bhoopal Singh Tomar, Shyam Sundar Dey, Jeetendra Kumar Ranjan, Narendra Singh, Narendra Kumar, Kamla Mahajani

**Affiliations:** ^1^ Division of Vegetable Science, ICAR-Indian Agricultural Research Institute, New Delhi, India; ^2^ ICAR-Indian Agricultural Statistical Research Institute, New Delhi, India; ^3^ Division of Plant Quarantine, ICAR-National Bureau of Plant Genetic Resources, New Delhi, India; ^4^ Centre For Protected Cultivation Technology (CPCT), ICAR-Indian Agricultural Research Institute, New Delhi, India; ^5^ Krishi Vigyan Kendra (KVK), Jhalawar, Agricultural University, Kota, Rajasthan, India; ^6^ ICAR-Indian Agricultural Research Institute, Hazaribag, India; ^7^ College of Community and applied Sciences, Maharana Pratap University of Agriculture and Technology, Udaipur, Rajasthan, India

**Keywords:** sponge gourd, *Tomato leaf curl New Delhi virus* (ToLCNDV), genotype by environment interaction, GGE biplots, GYT biplot

## Abstract

*Tomato leaf curl New Delhi virus* (TolCNDV) causes yellow mosaic disease, which poses a significant biotic constraint for sponge gourd cultivation, potentially resulting in crop loss of up to 100%. In the present investigation, 50 diverse genotypes were screened for 3 years under natural epiphytotic conditions. A subset of 20 genotypes was further evaluated across four different environments. The combined analysis of variance revealed a significant genotype × environment interaction. Eight genotypes consistently exhibited high and stable resistance in the preliminary screening and multi-environment testing. Furthermore, genotype plus genotype × environment interaction biplot analysis identified DSG-29 (G-3), DSG-7 (G-2), DSG-6 (G-1), and DSGVRL-18 (G-6) as the desirable genotypes, which have stable resistance and better yield potential even under diseased conditions. The genotype by yield × trait biplot analysis and multi-trait genotype–ideotype distance index analysis further validated the potential of these genotypes for combining higher yield and other desirable traits with higher resistance levels. Additionally, resistant genotypes exhibited higher activities of defense-related enzymes as compared to susceptible genotypes. Thus, genotypes identified in our study will serve as a valuable genetic resource for carrying out future resistance breeding programs in sponge gourd against ToLCNDV.

## Introduction

Sponge gourd (*Luffa cylindrica* Roem., 2n = 2x = 26) is a monoecious and annual cucurbitaceous vegetable, which is a native of the subtropical Asian region, particularly India (Kalloo, 1993). It is commonly grown for its tender fruits, natural sponge, and potential medicinal properties. Based on varied utilizations, it is vernacularly known as dishcloth gourd, towel gourd, vegetable sponge, and smooth gourd (Porterfield, 1955). Its tender fruits are a rich source of antioxidants, *viz*., vitamin A and vitamin C, flavonoids, and minerals Mg, K, Fe, Ca, Cu, Zn, Na, and Mn (Oboh and Aluyor, 2009; Azeez et al., 2013.).

**Figure 1:**
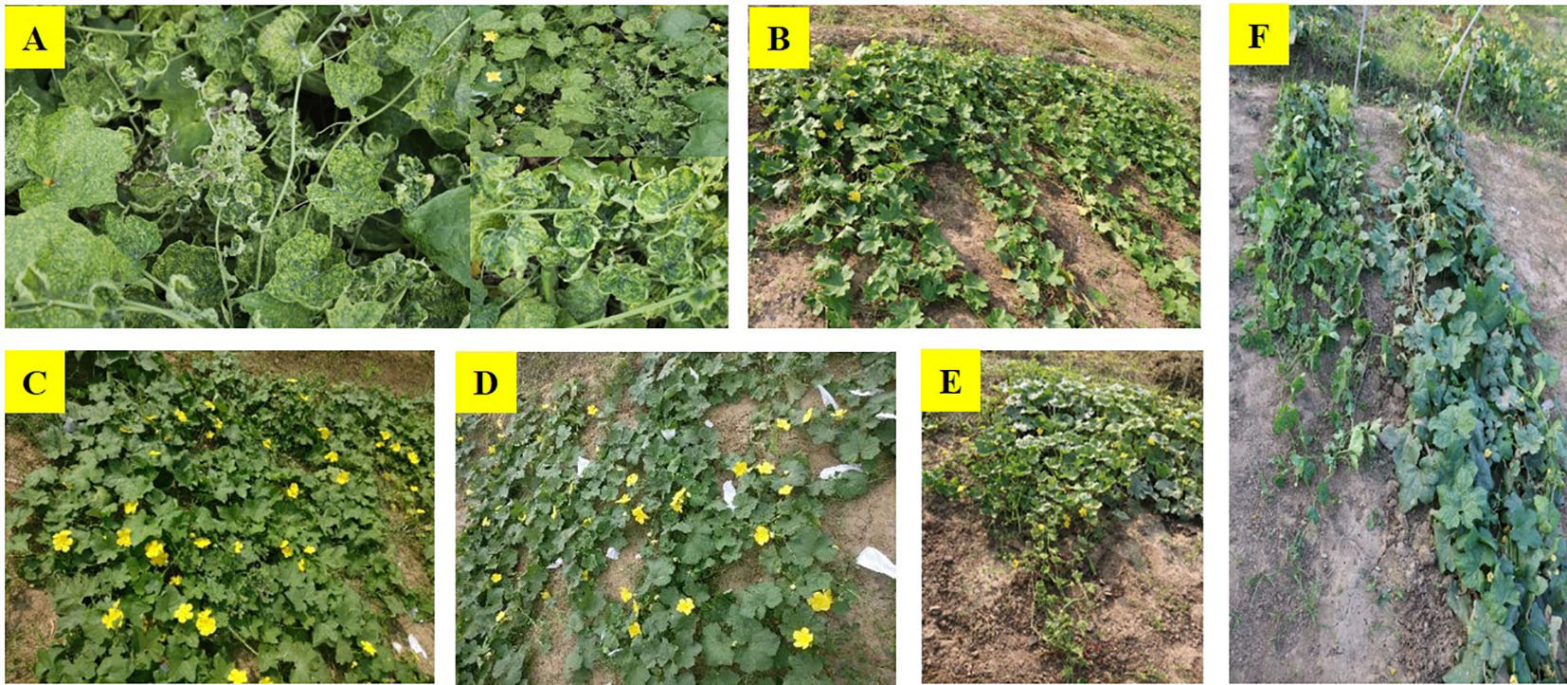
**(A)** Symptoms of ToLCNDV, severe mosaicism with chlorotic leaves, upward curling of upper leaves, shortened internodes, small, distorted leaves; **(B)** DSG-6(G-1), resistant; **(C)** DSG-29(G-3), resistant; **(D)** DSG-7(G-2), resistant; **(E)** Pusa Supriya(G-49), susceptible; **(F)** plants of DSG-29(R) and susceptible genotype at 50 days after sowing (Sept 2021).

Sponge gourd is cultivated during the spring–summer and *kharif* seasons in Northern India and throughout the year in Southern India (Islam et al., 2011; Singh et al., 2017; Singh et al., 2019a, b; Nagar et al., 2023). *Tomato leaf curl New Delhi virus* (ToLCNDV) is significantly impacting its cultivation, leading to a complete yield loss (100%) under epidemic conditions (Sohrab et al., 2003; Islam et al., 2010, 2011). Plants which are susceptible to ToLCNDV exhibit yellow spots on new leaves, leading to severe mosaicism with chlorotic leaves, upward curling of upper leaves, and shortened internodes (**Figure 1**). Furthermore, severe infestation leads to the development of small and distorted leaves with misshapen fruits (Sohrab et al., 2003). ToLCNDV is a bipartite begomovirus belonging to the Geminiviridae family which is transmitted naturally by an insect vector, white fly (*Bemisia tabaci*), in a circulative and persistent manner (Zaidi et al., 2017). At present, we rely only on insecticides for controlling the insect vector, thereby increasing the financial pressure on resource-poor farmers, as sponge gourd is mainly cultivated by small and marginal farmers in India and other developing countries. Therefore, the best alternative would be cultivating a resistant/tolerant variety, which is not only a simple, effective, economical, eco-friendly, but also most practical approach. However, this idea of having a resistant/tolerant variety is far from being a reality owing to the slow advancement in resistance breeding research in sponge gourd due to limited information on the inheritance and scarcity of stable resistance sources. Furthermore, host resistance involves a complex network of molecular and biochemical events that determines susceptibility or resistance. The resistance to diseases in plants is associated with the activation of a wide range of host defense mechanisms, including both preexisting physical and chemical barriers and inducible responses that disrupt pathogen establishment (Jones and Dangl, 2006; Zhao et al., 2008; Vanitha et al., 2009). Understanding these mechanisms is crucial for identifying effective and durable resistant sources. The accumulation and activity of enzymes such as catalase, phenyl ammonia lyase, super oxidase dismutase, peroxidase, hydrogen peroxide, and total phenol contribute in providing defense against viral infections and insect infestation (Quecini et al., 2007; Dieng et al., 2011). Thus, differential plant enzymatic activities in plants help in identifying and distinguishing between the resistant and susceptible genotypes against ToLCNDV.

The success of resistance breeding programs relies on the identification of stable resistant sources that consistently perform well across environments. To receive a breakthrough in this aspect, it is imperative to understand the role of independent and collective influences of genotypes and environmental factors. For this purpose, researchers have widely embraced various multivariate techniques and recent approaches like the Genotype plus Genotype × Environment Interaction (GGE) biplot to assess the significance of Genotype × Environment Interaction (GEI). The GGE biplot is a graphical representation methodology that employs indirect selection, effectively removing the main environmental effects and considering only the genotypic main effects and GEI in multilocational trials (Yan et al., 2000; Yan and Kang, 2003; Parihar et al., 2017; Singh et al., 2020; Sankar et al., 2021). The genotype by yield × trait (GYT) biplot is used to address the problem of combining yield with other important traits and aids in genotype evaluation for multiple traits (Yan and Frégeau-Reid, 2018; Merrick et al., 2020; Ebrahimi, 2023). The utilization of the Multi-trait Genotype-Ideotype Distance Index (MGIDI), which helps in effective and accurate selection of superior genotypes based on the average performance of multiple traits, overcomes the limitation of collinearity of linear selection indexes (León et al., 2021; Olivoto and Nardino, 2021; Pour-Aboughadareh et al., 2021; Uddin et al., 2021; Farhad et al., 2022; and Osuna-Caballero et al., 2022).

However, there is lack of information on identification of stable ToLCNDV-resistance sources using multi-environment data in sponge gourd. As ToLCNDV is endemic at the Indian Agricultural Research Institute, thus the endemic pressure at this site provides the opportunity to carry out a resistance breeding program under natural screening. Therefore, the present study was aimed with the following objectives: (i) to identify a stable resistant source against ToLCNDV in sponge gourd, (ii) to identify genotypes having the capability to combine higher yield potential and other desirable economical traits with higher resistance level, and (iii) to identify the ideal test environment that supports natural screening for resistance breeding program in sponge gourd.

## Materials and methods

### *Plant material for* multi-environmental field trials

In preliminary screening, 50 genotypes of sponge gourd (**Supplementary Table 1**), including inbred lines, advanced breeding lines, released cultivars, and germplasm accessions, were evaluated for ToLCNDV disease during the *kharif* season of 2018, 2019, and 2020. The experiment was conducted at Research Farm, Division of Vegetable Science, ICAR-IARI, New Delhi, in a randomized complete block design with three replications. Based on 3-year data of screening, a subset of 20 genotypes consisting of identified resistant genotypes, released varieties, and promising genotypes were further evaluated against ToLCNDV in three replications across four different environments during 2021 (**Table 1**). The environments consisted of two distinct seasons along with four locations, *viz*., Research Farm, Division of Vegetable Science during the *Kharif* season (E1), CPCT Farm during the *Kharif* season (E2), Jhalawar during the *Kharif* season (E3), and Research Farm at Division of Vegetable Science during the spring–summer season (E4). A spreader row of ToLCNDV-susceptible check (DSG-55) was planted after every third row of the test populations to ensure adequate disease pressure under natural conditions in all field trials at each environment. One row of susceptible check was also planted on all the sides of the experimental field. The recommended package of practice was followed in each environment, with the exception that no insecticides were sprayed to prevent the reduction in white fly proliferation. The crops were sown in rows of 2.5 m with 75-cm spacing between the plants.

**Table 1:** List of sponge gourd genotypes evaluated against *ToLCNDV* resistance across environments.

S. no	Genotypes	Genotype code	Fruit color	Vulnerability index at individual environments								Category
				RFDVSK-18	RFDVSK-19	RFDVSK-20	RFDVSK-21	CPCTK-21	JHK-21	RFDVSS-21	Overall mean	
1	DSG-6	G-1	Dark green	4.44	4.59	4.30	4.90	4.96	3.20	0.59	3.85	R
2	DSG-7	G-2	Light green	3.04	3.41	3.26	2.77	3.11	0.89	0.26	2.39	R
3	DSG-29	G-3	Dark green	7.41	8.89	7.70	8.67	7.52	4.43	1.02	6.52	R
4	DSGVRL-22	G-4	Green	10.74	11.11	11.70	12.96	10.52	4.28	1.95	9.04	R
5	DSGVRL-23	G-5	Dark green	15.80	15.90	13.70	15.52	13.65	6.33	4.16	12.15	R
6	DSGVRL-18	G-6	Dark green	19.26	18.63	19.84	19.90	18.80	10.70	3.65	15.83	R
7	DSGVRL-3	G-7	Dark green	21.73	23.41	20.43	23.78	21.17	19.83	5.76	19.44	R
8	DSGVRL-2	G-8	Dark green	22.95	26.59	23.26	25.01	21.73	21.80	6.06	21.06	R
9	DSGVRL-4	G-9	Dark green	27.91	26.74	25.63	29.11	27.57	22.65	6.22	23.69	R
10	DSG-43	G-20	Dark green	52.59	54.07	52.89	52.89	55.74	55.12	12.47	47.97	MR
11	DSG-31	G-22	Light green	54.96	56.44	58.37	58.37	56.49	54.64	14.81	50.58	MS
12	KSG-14	G-29	Dark green	72.30	72.00	70.81	70.81	69.20	56.98	28.69	62.97	MS
13	Pusa Sneha	G-35	Dark green	74.52	79.41	76.59	78.17	53.28	60.02	19.34	63.05	MS
14	CHSG-1	G-39	Green	83.89	81.78	80.74	80.74	83.40	74.13	25.22	72.84	MS
15	CHSG-2	G-40	Green	82.22	83.41	81.19	81.19	80.84	69.66	33.50	73.14	MS
16	JSLG-55	G-42	Light green	85.33	84.30	84.07	84.07	83.28	67.47	28.89	73.92	MS
17	PSG-9	G-46	Green	90.52	92.15	90.81	90.81	91.46	67.57	48.20	81.65	S
18	DSG-55	G-48	Light green	94.67	95.56	94.96	94.81	94.96	86.17	31.00	84.59	S
19	Pusa Supriya	G-49	Light green	95.26	96.30	94.96	95.80	85.65	74.72	28.06	81.54	S
20	Kalyanpur Hari Chikni	G-50	Dark green	96.59	95.41	96.30	95.36	96.59	78.25	29.93	84.06	S

RFDVSK-18, Research Farm of Division of Vegetable Science, IARI during the kharif season of 2018; RFDVSK-19, Research Farm of Division of Vegetable Science, IARI during the kharif season of 2019; RFDVSK-20, Research Farm of Division of Vegetable Science, IARI during the kharif season of 2020; RFDVSK-21, Research Farm of Division of Vegetable Science, IARI during the kharif season of 2021; CPCTK-21, Centre for Protected Cultivation Technology farm during the Kharif season of 2021; JHK-21, Jhalawar during the Kharif season of 2021; RFDVSS-21, Research Farm of Division of Vegetable Science, IARI during the spring–summer season of 2021; R, resistant; MR, moderately resistant; MS, moderately susceptible; S, susceptible.

### Disease scoring and vulnerability index estimation

Plants were scored after 60 days of sowing using a six-point scale from 0 to 5 (Sohrab, 2005; Islam et al, 2011) as per the ToLCNDV disease symptoms. The scoring criteria were as follows: 0 = absence of symptoms; 1 = mild mosaic on young leaves covering more than 10% of area; 2 = mosaic on young leaves covering over 25% of the area; 3 = mosaic on young leaves covering more than 50% of the area, along with blistering and puckering of leaves; 4= mosaic on young leaves covering more than 75% of the area, resulting in distorted leaves; and 5 = mosaic on young leaves covering more than 75% of the area accompanied by distorted leaves and stunted plant growth. The vulnerability index (VI) values for each genotype were determined by scoring individual plants based on the presence of *ToLCNDV* disease symptoms. The VI values were calculated by degree of resistance among each genotype, using an equation outlined by Silbernagel and Jafari (1974) and modified by Bos (1982).


$$VI=\left(\frac{{0n}_{0}+{1n}_{1}+{2n}_{2}+{3n}_{3}+{4n}_{4}+{5n}_{5}}{nt(nc-1)}\right)\times 100$$

where,

*n_0_*, *n_1_*,…*n_5_* = number of plants in each score category (0–5),

*nt* = total number of plants, and

*nc* = total number of categories

Genotypes were categorized into five groups based VI values (Havey, 1996; Islam et al., 2011):

VI = 0—immune
VI = 1%–25%—resistant
VI = 26%–50%—moderately resistant
VI = 51%–75%—moderately susceptible
VI = 76%–100%—susceptible

### Observations

During 3 years (2018–2020) of preliminary screening, observations were also recorded for eight quantitative traits, *viz*., days to first male flower anthesis (DFMA), days to first female flower anthesis (DFFFA), days to first fruit harvest (DFFH), average fruit weight (AFW), fruit length (FL), fruit diameter (FD), number of fruits per plant (FPP), and total fruit yield per plant (FYLP) along with the vulnerability index under ToLCNDV incidence. Based on correlation results of quantitative traits with VI values, four quantitative traits, *viz*., days to first female flower anthesis, average fruit weight (g), number of fruits per plant, and total fruit yield per plant (kg/plant), were recorded for 20 genotypes during multi-environment testing during 2021.

### Construction of the Genotype plus Genotype × Environment Interaction biplot

The genotype (G) and genotype by environment (GE) interaction can be graphically represented by the GGE biplot based on the scores of first two principal components (PC) resulting from the singular value decomposition (SVD) (Yan et al., 2000; Yan, 2002; Yan and Hunt, 2001; Yan and Kang, 2003).


$$Y_{ij}=\;µ+\;e_{j}+\;\sum_{n=1}^{N}\lambda_{n}\gamma_{in}\delta_{jn}+\varepsilon_{ij}$$

where Y_ij_ = mean response of the ith genotype (i = 1,…,i) in the j^th^ environment (j = 1,., j); µ = grand mean; e_j_ = environment deviations from grand mean; λ_n_ = eigenvalue of PC analysis axis; γ_in_ and δ_jn_ = genotype and environment PC scores for axis n; N = number of PCs retained in model; ϵ_ij_ = residual effect ~N (0, σ^2^e).

### Data analysis and software

The GGE biplot, GT biplot, and GYT biplot were created utilizing GGE biplot software in R. The correlations were determined using the Pearson correlation coefficient method and plotted with the GGally package of R. The cluster heat map was performed using the “heatmap.2” function through the plots v3.0.1.1 library implemented in R. The multi-trait genotype–ideotype distance index (MGIDI) was performed using the “metan” package in R software (Olivoto and Nardino, 2021).

### Biochemical characterization

#### Sample collection and storage

The activity and accumulation of defense-related enzymes with level of resistance against ToLCNDV in sponge gourd were assessed. Eight resistant genotypes, four genotypes each of moderately resistant, moderately susceptible, and susceptible category, were subjected for biochemical characterization. The fresh leaf samples were collected 60 days after sowing, when plants were fully infected with ToLCNDV, and stored at −20°C until the biochemical tests were performed. For catalase activity, peroxidase activity, total phenol content, and total soluble protein content, 1 g of leaves was added with 10 ml of 0.1 M phosphate buffer in a mortar and pestle. The mixture was crusted until a slurry was obtained. Similarly, 0.5 g of leaves was crushed in mortar and pestle for hydrogen peroxide analysis and 0.5 ml of 0.5 mM TCA acid and 1 M pH 8 phosphate buffer were used to prepare a slurry. 2 ml of each homogenates was taken in tubes and centrifuged at 10,000 rpm for 15 min. The supernatant was taken out for further analysis of individual biochemical parameters.

#### Total soluble protein content

It was estimated using the protocol outlined by Bradford (1976). 140 μl of Bradford reagent was added with 60 μl plant extract till 20 samples were completed, and absorbance reading was taken at 600 nm.

#### Phenol assay

The total phenol content was determined using the Folin–Ciocalteu reagent. 2 ml of plant extract was added with 2 ml of absolute ethanol in a test tube and boiled until all ethanol evaporated, leaving only the plant residue. 5 ml of distilled water was added to the plant residue, and phenol solution was prepared by mixing 2 ml FC reagent and 4 ml of 35% sodium carbonate. The 150 μl of solution was added in wells with 50 μl of diluted plant residue, and a reading of absorbance was taken at 650 nm after incubating for 1 h.

#### Hydrogen peroxide assay

2 ml of 1 M potassium iodide was added with 1 ml of 0.1 M phosphate buffer for master mix preparation. 150 μl of mix was added along with 50 μl of hydrogen peroxide sample extract, and reading was taken at 390 nm after 20 min of incubation at room temperature.

#### Peroxidase assay

The peroxidase activity was estimated using the Chance and Maehly (1955) method. The master mix was prepared by adding 192 μl of 1 M guaiacol, 24 μl hydrogen peroxide, and 11.38 ml of 50 mM phosphate buffer. 150 μl of master mix was added along with 50 μl of plant extract in each well of the ELISA plate till 20 samples were completed, and absorbance at 450 nm was measured before and after 1 min.

#### Catalase assay

Catalase activity (CAT) was assessed using the Zhang et al. (2007) protocol. The solution for catalase activity included 11.6 ml of 0.1 M pH 7 phosphate buffer and 140 μl of hydrogen peroxide. 175 μl of solution was added to ELISA plate well, and 25 μl of plant extract until all the samples were added. The absorbance reading was taken at 270 nm for 3 min at 30-s intervals.

#### Superoxidase dismutase (SOD)

1 g of the leaf sample was mixed with 10 ml of potassium phosphate buffer (50 mM) pH 7 solution in a mortar and pestle to prepare the slurry. Then, the homogenate was centrifuged for 10 min at 10,000–12,000 rpm and the supernatant solution was take out into another tube. 1,100 μl potassium phosphate buffer (50 mM) was mixed with pH 7, 372.20 μl EDTA (0.1 mM), 105.99 μl sodium carbonate (50 mM), 149.21 μl, L-methionine (SOD enzyme protector), 817.64 μl NBT (50 mM), and 376.36 μl riboflavin (O-2 generator): in a tube and form a 3 ml master mix. 10 μl of supernatant was added to an ELISA plate well along with 190 μl of master mix, and absorbance was read at 560-nm wavelength with the help of a spectrophotometer.

#### Phenyl ammonia lyase (PAL)

The PAL activity was assessed following the procedure outlined by Zucker (1965). The homogenate was prepared in a chilled pestle and mortar by crushing 0.5 g of lead sample with 5 ml of sodium borate buffer (0.1 M) pH 8.5 solution. The slurry was centrifuged in a 2-ml tube at 10,000–12,000 rpm for 10 min. The supernatant was transferred to another 2-ml tube. 62.5 µl enzyme extract, 800 µl of sodium borate buffer, and 700 µl of (12 mM) phenylalanine were added in each test tube and incubated at 40°C in a water bath for 1 h. The reaction was halted by adding 200 µl of 5 N HCl, followed by the addition of 0.5 ml of 0.1 M Trans-cinnamic acid (TCA). The 62.5 µl enzyme extract and 800 µl of sodium borate buffer were added in each test tube, along with 700 µl of (12 mM) phenylalanine, and tubes were incubated at 40°C in a water bath for 1 h. 200 µl of 5 N HCl was added to stop the reaction. Then, 0.5 ml of trans-cinnamic acid (TCA) (1 M) was added, and the light absorbance was estimated at 290 nm. The absorbance reading was measured at 290 nm.

## Results

### Identification of genotypes for multi-environment testing

#### Disease reaction of genotypes against ToLCNDV

There were 50 diverse germplasms including commercial varieties and advanced breeding lines that were subjected to three consecutive years of natural screening in the *kharif* season. The pooled vulnerability index score of 3 years varied from 3.23% to 96.10% (**Supplementary Table 1**). Eight genotypes showed resistant reaction against ToLCNDV with the lowest VI value, which was recorded in DSG-7 (3.23%), followed by DSG-6 (4.44%) and DSG-29(8.00%). There were 11 genotypes that showed a moderately resistant type of reaction, whereas 14 showed moderately susceptible reaction. There were 17 genotypes including all the commercially grown varieties that were found to be susceptible with VI values ranging from 76.50% to 96.10% against ToLCNDV. Furthermore, susceptible check DSG-55 showed a pooled mean VI value of 95.06%, thus indicating high disease pressure across the years of preliminary screening.

#### Evaluation of genotypes based on mean vs. stability biplot

The average environment coordination (AEC) view of the mean vs. stability biplot based on genotype-focused singular value portioned (SVP = 1) was conducted to evaluate the mean performance and stability of 50 genotypes across the tested environments (**Supplementary Figure 1**). The mean vs. stability biplot revealed that genotypes, DSG-7(G-2), DSG-6(G-1), DSG-29(G-3), DSGVRL-22(G-4), DSGVRL-23(G-5), DSGVRL-18(G-6), DSGVRL-3(G-7), and DSGVRL-2 (G-8), were categorized as stable resistant genotypes against ToLCNDV over the 3 years. These genotypes were positioned in a descending order with respect to farthest genotype at the left side on the AEC abscissa from the biplot origin, expressing low VI values. Moreover, all these genotypes were present on the AEC ordinate, thus showing resemblance in their response toward disease severity against ToLCNDV. These genotypes, except DSGVRL-22(G-4) and DSGVRL-3(G-7), showed stability regarding the number of fruits and fruit yield per plant. DSG-29(G-3), DSG-7(G-2), and DSGVRL-23(G-5) were most desirable with respect to earliness *viz*., DFMA, DFFFA, and DFFH. Regarding fruit length, DSG-512 (G-30) and DSG-51-1 (G-47) had higher and stable performance whereas DSG-7(G-2), DSGVRL-22(G-4), and DSGVRL-3(G-7) had higher and stable performance for fruit diameter. Similarly, DSG-512 (G-30) was the most ideal and stable for average fruit weight during all the years.

#### Correlation between VI values and yield components

**Figure 2:**
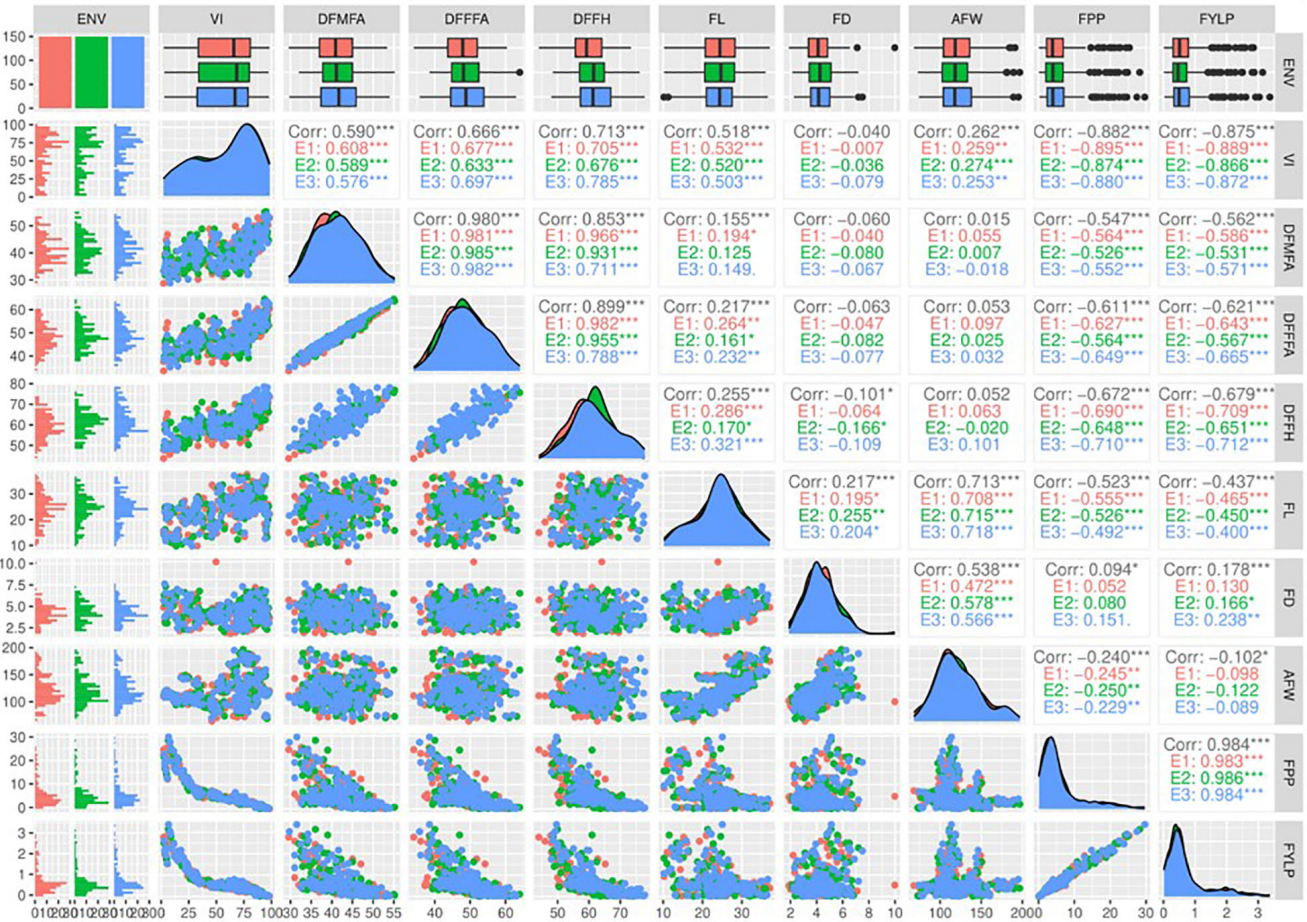
Scatter plot matrix showing the correlation between nine agro-morphological traits of the sponge gourd genotypes evaluated under *ToLCNDV* conditions during the *kharif* season for 3 years (2018–2020). VI, vulnerability index; DFMFA, days to first male flower anthesis; DFFFA, days to first female flower anthesis; DFFH: days to first fruit harvest; FL, fruit length (cm); FD, fruit diameter (cm); AFW; average fruit weight (g); FPP, number of fruits per plant; FYLP, fruit yield per plant (kg/plant). *** p-value< 0.001, ** p-value< 0.01, * p-value< 0.05.

Correlation analysis revealed that 30 pairs of nine traits evaluated were significantly correlated considering all the three harvesting seasons (**Figure 2**). Among them, 27 pairs exhibited significant correlation at a significance level of p< 0.001, whereas three pairs were significant at p< 0.05. The vulnerability index showed a highly significant positive correlation with DFMA (r = 0.59), DFFFA (r = 0.666), and DFFH (r = 0.713). In contrast, it had a highly significant negative correlation with fruit yield per plant and number of fruits per plant. In addition, all these traits showed a similar trend for all 3 years. Fruit yield and number of fruits per plant exhibited the highest positive correlation of r = 0.984 with similar results for all the three harvesting seasons. Furthermore, the yield is negatively correlated with flowering traits, viz., DFMA (r = −0.562), DFFFA (r = −0.621), and DFFH (r = −0.679). This negative correlation suggests that earliness leads to a higher yield in genotypes screened against ToLCNDV. The significant positive correlation of the vulnerability index with flowering traits indicates that high VI values increase the duration of appearance of first male and female flowers and, ultimately, the harvesting of first fruits. Thus, susceptibility against ToLCNDV delays the flowering. Similarly, VI showed a significant negative correlation with fruit number and fruit yield per plant, severely affecting yielding capacity of the genotypes under ToLCNDV infection in sponge gourd.

#### Cluster analysis

**Figure 3:**
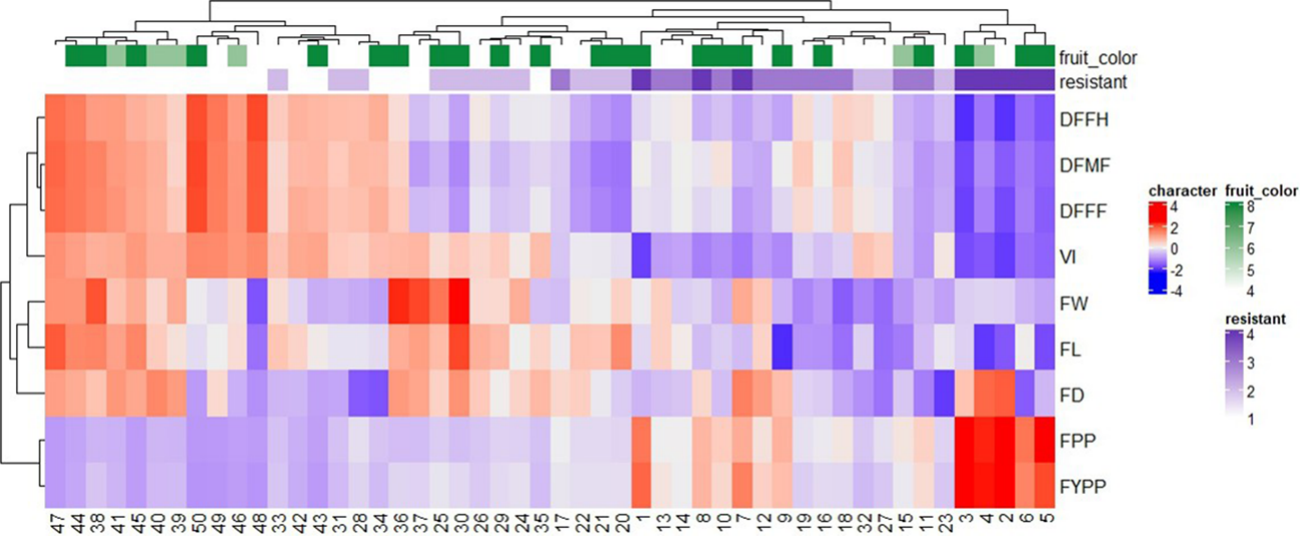
Dendrogram along with heat-map of 50 sponge gourd germplasms generated by performing hierarchical cluster analysis based on 3-year (2018–2020) performance. Each row represents different quantitative traits, and each column represents a genotype. The higher the trait value, the brighter is the red and similarly, the lower the trait value, the brighter is the blue color. Traits code: DFFF, days to first female flower anthesis; DFMF, days to first male flower anthesis; DFFH, days to first fruit harvest; VI, vulnerability index; FW, average fruit weight (g); FL, fruit length (cm); FD, fruit diameter (cm); FPP, number of fruits per plant; FYPP, fruit yield per plant (kg/plant). Number corresponds to genotypes as listed in **Supplementary Table 1**.

The heatmap and hierarchical clustering based on earliness, yield and its attributing components, and ToLCNDV vulnerability index were carried out to show a chromatic evaluation of genotypes (**Figure 3**). The heat map analysis produced two dendrograms; the one in the horizontal direction represents the genotypes, and the second in the vertical direction represents the traits influenced by this diffusion. The relationship and strength among variables and genotypes determines the color of each box. The genotypes were grouped to identify genotypes with common characteristics in terms of resistance, earliness, and higher yield. A clustered heat map distinguished the genotypes into seven groups based on resistance level and yield-related traits. Group I consisted of five genotypes (G-2, G-3, G-4, G-5, and G-6), exhibiting resistance with low VI values, higher yield traits, and earliness. Group II included eight genotypes, mostly moderately resistant and three moderately susceptible genotypes based on yield and other traits. Group III comprised eight genotypes, two as resistant and the remaining moderately resistant. The resistant check (DSG-6) belonged to this cluster based on fruit-related traits, delayed anthesis, and harvesting time. Furthermore, Groups IV, V, VI, and VII consisted of 8, 4, 10, and 7 genotypes, respectively, and were classified as moderately susceptible and susceptible based on their high VI values.

### Multi-location evaluation of genotypes

#### Analysis of variance and mean performance

Considering 3-year screening results, 20 diverse genotypes, including identified resistant genotypes, commercially grown varieties, and promising genotypes, were evaluated across four diverse environments during 2021 (**Table 1**). Significant genotypic variations (P<0.001) were observed through individual environment ANOVA for traits such as vulnerability index against ToLCNDV, days to first female flower anthesis, number of fruits per plant, average fruit weight, and fruit yield under disease incidence during 2021. Combined ANOVA also revealed significant genotypic differences, as well as significant environment and genotype × environment interaction effects for all the traits including vulnerability index (**Table 2**). The contribution of genotypic variance was recorded higher as compared to GEI and environmental variance for all the traits.

**Table 2:** Analysis of variance for the evaluated parameters of 20 sponge gourd genotypes tested in four environments.

Source of variation	DF	Traits														
		VI			DFFFA			AFW			FPP			FYPP		
		MS	F value	Pr > F	MS	F value	Pr > F	MS	F value	Pr > F	MS	F value	Pr > F	MS	F value	Pr > F
GEN	19	9,654.32	607.62	<.0001	271.51	33.78	<.0001	1,054.14	19.59	<.0001	309.18	105.37	<.0001	4.19	96.95	<.0001
REP	2	0.46	0.03	0.9715	21.08	2.62	0.0758	47.71	0.89	0.4141	1.41	0.48	0.6197	0.02	0.55	0.5781
ENV	3	15,295.69	962.67	<.0001	365.33	45.45	<.0001	3,086.32	57.36	<.0001	581.16	198.07	<.0001	8.10	187.14	<.0001
GEN*ENV	57	363.85	22.90	<.0001	45.91	5.71	<.0001	388.08	7.21	<.0001	33.90	<.0001	11.55	0.41	9.49	<.0001
Error	158	15.889			8.038			53.81			2.934			0.043		

VI, vulnerability index; DFFFA, days to first female flower anthesis; AFW, average fruit weight (g); FPP, number of fruits per plant; FYPP, fruit yield per plant (kg/plant).

#### Identification of ideal genotypes based on mean vs. stability

**Figure 4:**
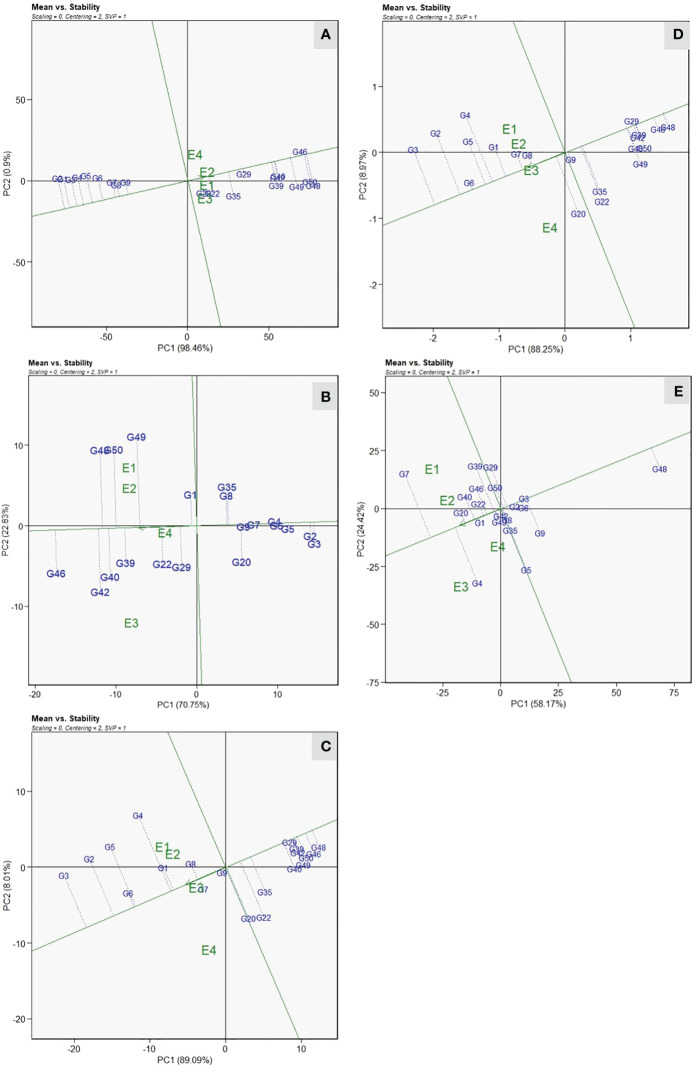
Mean vs. stability view of the GGE biplot of 20 sponge gourd genotypes across four testing environments for vulnerability index **(A)**, days to first female flower anthesis **(B)**, number of fruits per plant **(C)**, fruit yield per plant **(D)**, and average fruit weight **(E)**. There was no transformation of data (transform = 0), and data were centered by means of the environments (centering = 2). The biplot was based on “row metric preserving” [singular value partition (SVP) = 1]. Numbers correspond to genotypes as listed in **Table 1**.

All eight resistant genotypes were positioned on the left side of the AEA abscissa, whereas the remaining genotypes were placed toward the direction of the AEA abscissa from the biplot origins (**Figure 4A**). The genotypes positioned toward the direction reveal higher VI values, which indicate susceptibility, whereas genotypes in the opposite direction show lower VI values, thus signifying resistant reaction. Genotypes G-48(DSG-55) and G-2(DSG-7) were positioned at two extremes of AEA abscissa, respectively, showing the highest and lowest values of VI across the environments. The stability of genotypes, which is represented by projection on the AEA abscissa, reveals that genotype DSGVRL-2(G-8), followed by DSG-7(G-2) and DSG-29(G-3), was the most stable across the environments studied. Therefore, genotype DSG-7 (G-2) was considered as the ideal genotype based on mean performance and stability. According to Yan and Tinker (2006), Euclidian distance was used to estimate the distance between genotypes; thus, genotypes closer to ideal genotypes are regarded as favorable. Therefore, genotypes DSG-6(G-1), DSG-29(G-3), DSGVRL-22(G-4), DSGVRL-23(G-5), DSGVRL-18(G-6), and DSGVRL-3(G-7), were considered as desirable and stable performers with respect to low VI values. For earliness observed by days to first female flower anthesis, genotypes, DSG-29(G-3) and PSG-9(G-46) displayed the lowest and highest values, respectively (**Figure 4B**). Genotype DSG-29(G-3) was considered as the most ideal and stable performer, followed by DSG-7(G-2), DSGVRL-23(G-5), and DSGVRL-18(G-6), across the environments. Similarly, genotypes DSG-29(G-3) and DSG-55 (G-48) were positioned at two extremes of AEA abscissa, indicating the highest and lowest values concerning the yield traits, *viz*., number of fruits per plant and fruit yield per plant (**Figures 4C, D**). Genotype DSGVRL-18(G-6) was the most ideal and stable, followed by DSG-29(G-3) and DSG-6(G-1). Genotype DSG-6(G-1) was observed as the most ideal genotype, followed by DSG-43(G-20) and CHSG-2(G-40) for average fruit weight (**Figure 4E**).

#### *Mega environment delineation and the “which*-won-where” view

**Figure 5:**
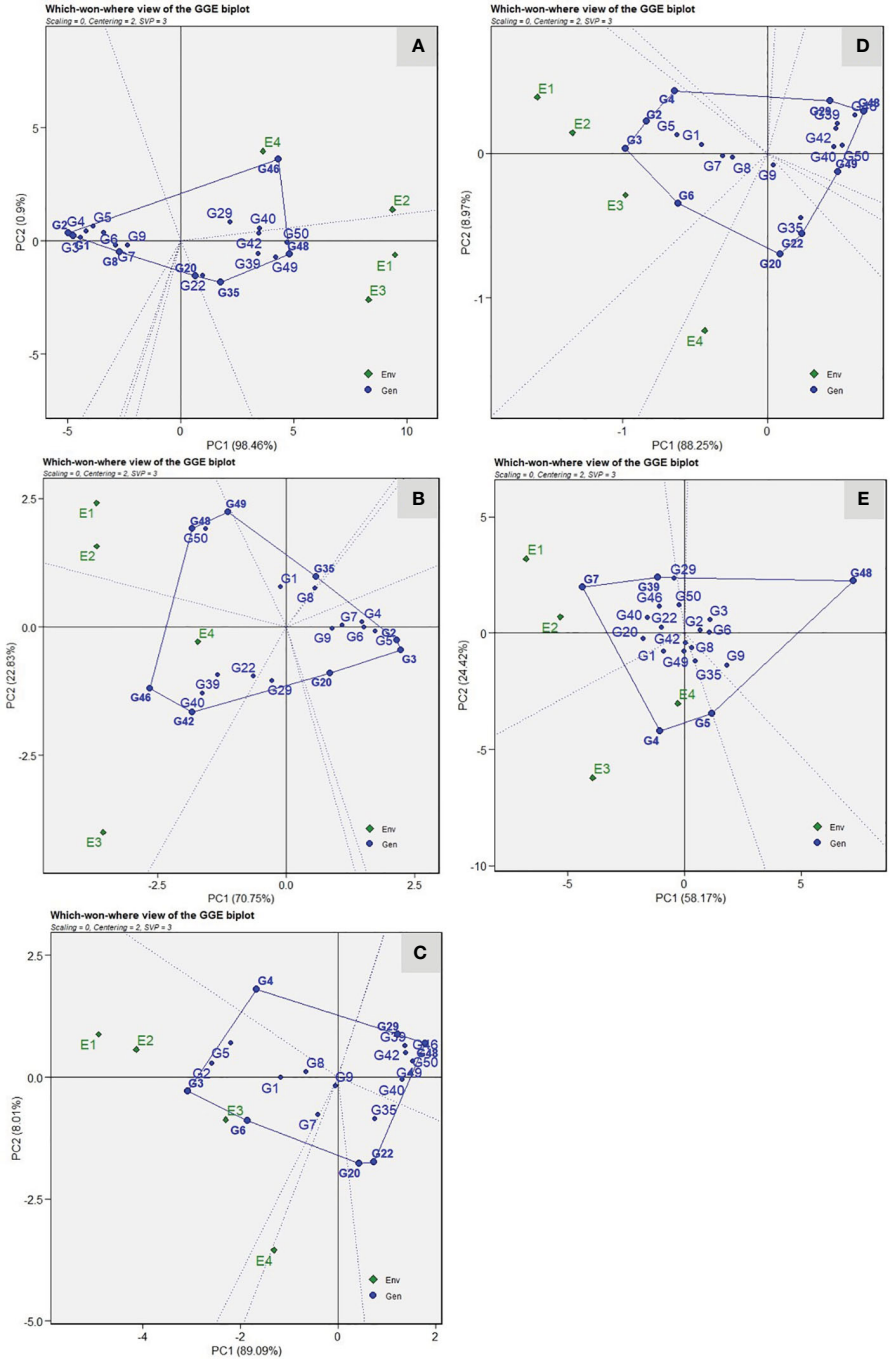
The polygon (which-won-where) view of the GGE biplot of 20 sponge gourd genotypes across four testing environments for vulnerability index **(A)**, days to first female flower anthesis **(B)**, number of fruits per plant **(C)**, fruit yield per plant **(D)**, and average fruit weight **(E)**. There was no transformation of data (transform = 0), and data were centered by means of the environments (centring = 2). The singular value is symmetrically partitioned into the genotype and the environment eigenvectors (SVP = 3). Numbers correspond to genotypes as listed in **Table 1**.

The “which-won-where” view of the GGE biplot revealed that resistant genotypes were positioned toward the left side of the origin and did not share the sector with any location. Thus indicating that their performance remained relatively consistent across the environments studied (**Figure 5**). For VI, the vertex genotypes, *viz*., DSG-7(G-2), PSG-9(G-46), Pusa Supriya(G-49), and Pusa Sneha(G-35), were considered as best or the poorest (**Figure 5A**). The test environments were grouped into two sectoral regions, *i.e*., mega environments. The first mega environment contains the test environment, E1 and E3, with a winning genotype, Pusa Supriya(G-49), which is susceptible to ToLCNDV due to high VI values. The second mega environment consists of test environments, E2 and E4, with winning genotype PSG-9(G-46), which is also susceptible, as indicated by high VI values. The genotypes DSG-7(G-2) and DSG-29(G-3), followed by DSG-6 (G-1), were plotted farthest on the left side, indicating the lowest VI scores across the environments. Genotypes DSG-29 (G-3), DSGVRL-23(G-5), Pusa Supriya(G-49), Kalyanpur Hari Chikni(G-50), PSG-9(G-46), and JSLG-55(G-42) were vertex genotypes indicating their best or poorest performance in the particular mega environment for days to first female flower anthesis (**Figure 5B**). E1 and E2 were present in one mega environment with the winning genotype Kalyanpur Hari Chikni(G-50), whereas genotype PSG-9(G-46) was observed as the winning genotype for the second mega environment, which consisted of E3 and E4. These genotypes were considered poor performers, as revealed by the delayed first female flower anthesis. However, genotypes DSG-29(G-3) and DSGVRL-23(G-5) presented on the extreme right side of the origin were the best performers with respect to earliness. The genotypes DSG-29(G3), DSGVRL-22(G-4), DSG-55(G-48), DSG-31(G-22), DSG-43(G-20), and DSGVRL-18(G-6) were vertex genotypes whereas environments E1, E2, and E3 were present in a single mega environment with DSG-29(G-3) as the winning genotype for number of fruits per plant (**Figure 5C**). For fruit yield per plant, genotypes DSG-29(G-3), DSGVRL-22(G-4), DSG-55(G48), DSG-43(G-20), and DSGVRL-18(G-6) were the vertex genotypes (**Figure 5D**). All the test environments are grouped in two sectors, thus indicating two mega environments. The first mega environment consisted of three test environments (E1, E2, and E3) and genotypes DSG-29(G-3), followed by DSG-7 (G-2), DSGVRL-22 (G-4), and DSGVRL-18 (G-6) were the best performers. Meanwhile, test environment E4 comes under the second mega environment, with genotype DSG-43(G-20) followed by DSG-31(G-22), which were considered as the best performers for that particular mega environment. In the case of average fruit weight, vertex genotypes were DSGVRL-3(G-7), DSGVRL-22(G-4), DSGVRL-23(G-5), DSG-55(G-48), and CHSG-1(G-39) (**Figure 5E**). The equality line divides the four test environments into two mega environments, E1 and E2 in the first and E3 and E4 in the second. DSGVRL-3(G-7) and DSGVRL-22(G-4) were the winning genotypes of the first and second mega environments, respectively.

#### Identification of ideal environment

**Figure 6:**
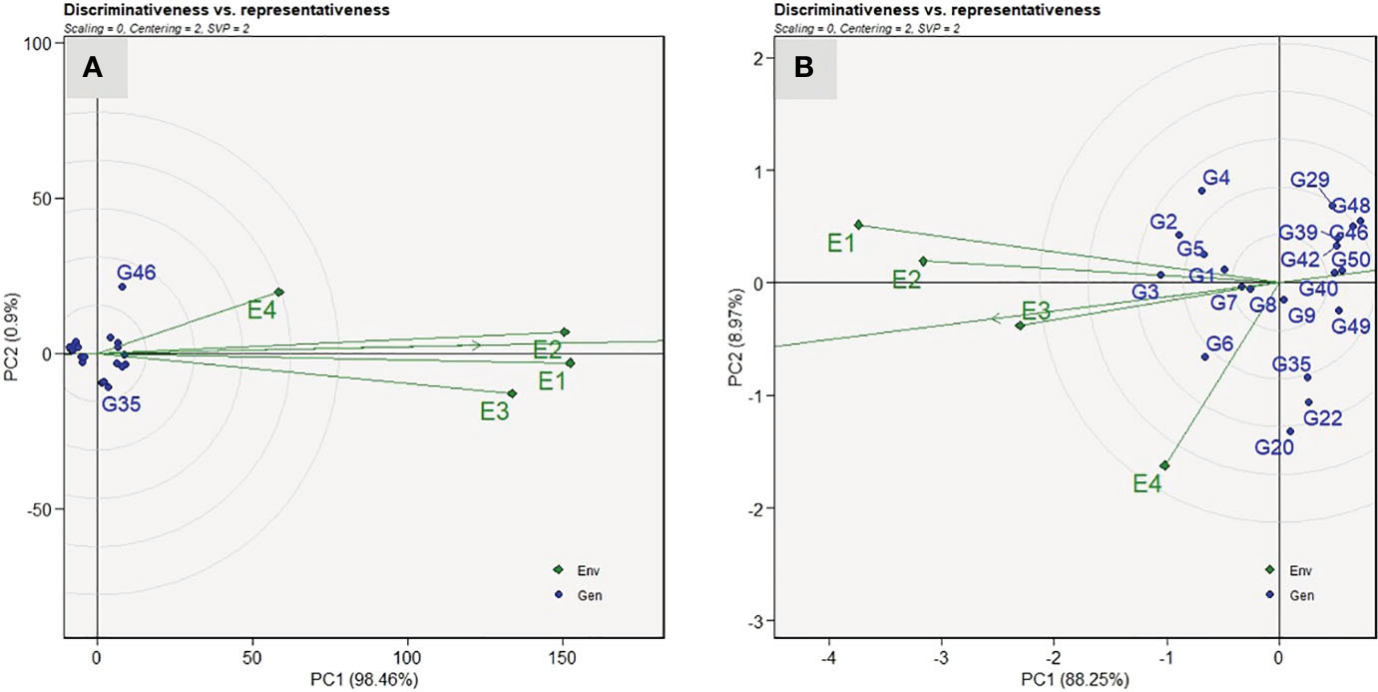
“Discrimitiveness vs. Representativeness” view of the GGE biplot of twenty sponge gourd genotypes across four testing environments for vulnerability index **(A)**, and fruit yield per plant **(B)**. There was no transformation of data (transform = 0), and data were centered by means of the environments (centring = 2). The biplot was based on “Column metric preserving.” (SVP = 2). Numbers correspond to genotypes as listed in **Table 1**.

Among the four test environments, the longest vector length was recorded in E1, followed by E2 and E3, whereas the shortest was in E4 for VI (**Figure 6A**). Thus, the test environment E1 was the most discriminating location for ToLCNDV screening compared with other locations, whereas E4 had less discriminating ability. Among the test environments, E1 and E2 were the most representative environments revealed by a small angle with AEA abscissa compared with other two environments for disease screening against ToLCNDV. The test environment E1 and E3 displayed acute angles with E2, thus indicating a close association and consensus on the genotypic response to ToLCNDV disease severity. Similarly, E1 was identified as the most discriminating location for fruit yield per plant, and E3 was the representative environment (**Figure 6B**).

#### Genotype selection utilizing genotype by trait and genotype by yield × trait biplots

**Figure 7:**
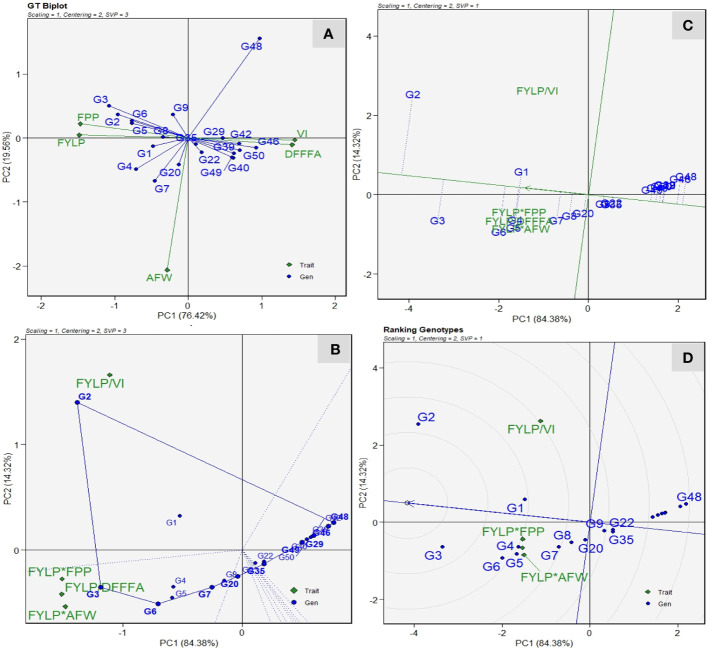
Genotype by trait (GT) and Genotype by yield × trait (GYT) biplots for twenty sponge gourd genotypes evaluated under ToLCNDV conditions. **(A)** GT biplot based on Scaling = 1, Centering = 2, and SVP = 3. **(B)** Which-won-where view of the GYT biplot based on Scaling = 1, Centering = 2, and SVP = 3. **(C)** Average Tester Coordination view of the GYT biplot based on Scaling = 1, Centering = 2 and SVP = 1. **(D)** Genotype ranking plot view of the GYT biplot based on Scaling = 1, Centering = 2, and SVP = 1. Trait codes are: - DFFFA: days to first female flower anthesis; VI: vulnerability index; AFW; average fruit weight (g); FPP: number of fruits per plant; FYLP: fruit yield per plant (kg/plant).

The GT biplot facilitates the depiction of relationships between genotype and traits based on trait-standardized data and singular value partitioning focused on traits. The GT biplot analysis showed a strong positive correlation with fruit yield and number of fruits per plant. However, both traits showed a negative correlation with vulnerability index and days to first female flower anthesis as indicated by an obtuse angle among them (**Figure 7A**). Similarly, we recorded a strong positive correlation between VI values and delayed flowering. These results are in conformity with the correlation graph of 3 years of preliminary screening. On the contrary, average fruit weight shows no relation with VI values. Genotype DSG-29(G-3) had a higher fruit yield and number of fruits per plant, low VI values, and earliness. However, DSG-55(G-48) had the highest VI values and delayed flowering. Genotypes DSGVRL-3(G-7) and DSGVRL-22(G-4) had high average fruit weight. Although the GT biplots reveal the association among the traits and genotypes’ trait profiles, they lack the practical utility in decision making on cultivar selection and rejection. Therefore, to address this, the proposed GYT biplot analysis was carried out (**Figure 7**). Higher days to first female flower anthesis and higher vulnerability index value are undesirable traits for sponge gourd improvement. Thus, the combination of yield*earliness (FYPP/DFFF) and yield*disease score (FYPP/VI) had a division operator (“/”). It reflects that their values were reversed before multiplication to the yield values. The which-where-won view helps in visualizing the genotypes’ trait profiles, and the perpendicular lines divide the yield-trait combinations into two sectors (**Figure 7B**). Genotype DSG-29(-3), followed by DSGVRL-18(G-6), had the largest values for FYLP/DFFF, FYLP*FPP, and FYLP*AFW, thus making them as the best genotype for combining fruit yield with earliness, number of fruits per plant and average fruit weight. Similarly, genotype DSG-7(G-2) had the highest level for FYLP/VI, indicating that it was superlative for combining fruit yield with a low vulnerability index. **Figure 7C** displays the superiority ranking of genotypes, along with their strengths and weaknesses, as assessed through the average tester coordinator (ATC) view of the GYT biplot. The average tester axis (ATA) is a single arrow line that passes through the biplot’s origin and signifies the direction of superior mean performance. Consequently, it aids in ranking genotypes according to their overall superiority. The genotypes were separated into better and worst performers based on the average overall performance by the perpendicular lines that pass through the biplot origin on ATA. It also suggests the balanced trait profile or strength and weakness for a trait or group of traits of genotypes. Thus, based on the average yield-trait combinations, the best-ranked genotype was DSG-7(G-2), followed by DSG-29(G-3), DSGVRL-18(G-6), DSGVRL-23(G-5), DSGVRL-22(G-4), and DSG-6(G-1), and so on as we move left to right on the ATA. Meanwhile, genotypes ranked poorest are plotted on the far right side of the ATA. The projection of genotypes suggests the strengths and weaknesses; thus, genotype DSG-7(G-2) has a strong performance level with regard to FYLP/VI, and DSG-29(G-3) was strong in FYLP/DFFF, FYLP*FPP, and FYLP*AFW, followed by DSGVRL-18 (G-6). However, the genotypes that are closer to ATA, *i.e*., short projection length, have a balanced trait profile; thus, DSG-29(G-3), followed by DSGVRL-18 (G-6) and DSGVRL-23 (G-5), had a more balanced overall trait profile. The ideal genotype was identified by genotype ranking plot view of the GYT biplot (**Figure 7D**). DSG-29(G-3) is located in the first concentric circle, thus making it an ideal genotype followed by DSG-7(G-2) and DSGVRL-18 (G-6) with less VI value, earliness, and higher yield.

#### Genotype selection using multi-trait genotype–ideotype distance index

**Figure 8:**
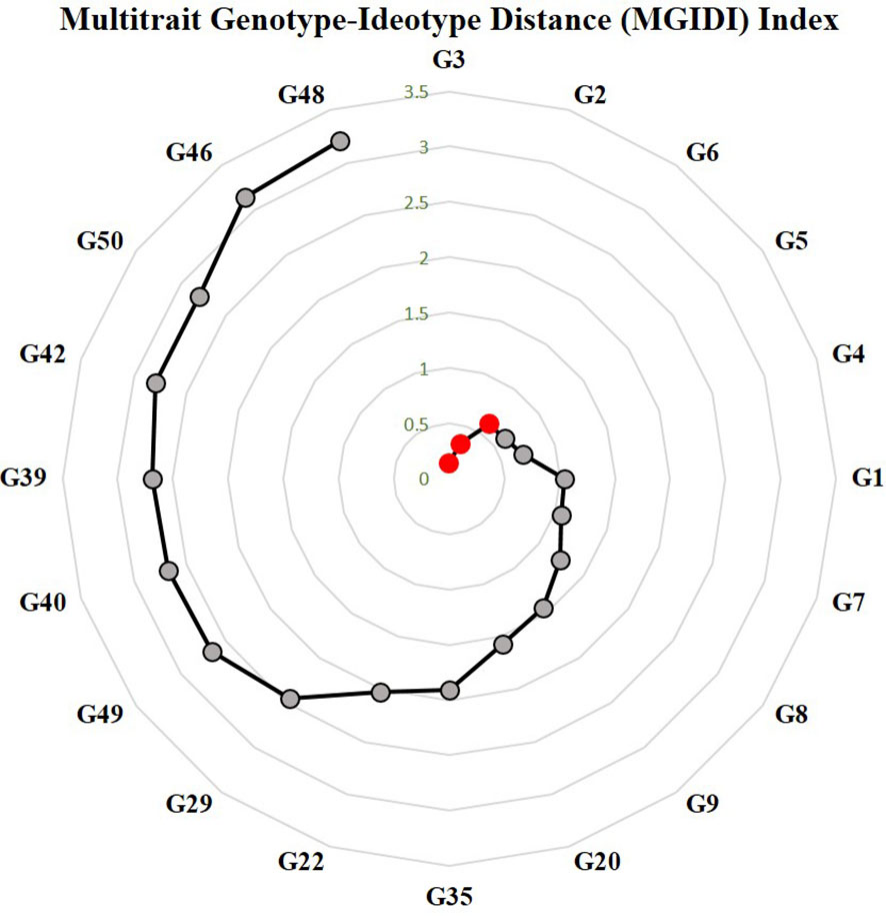
Genotype ranking in ascending order of the MGIDI index. The selected genotypes are shown in red circle. Numbers are correspond to genotypes as listed in **Table 1**.

The MGIDI relies on evaluating the distance between genotypes and specified ideotype aligned with the breeders’ preference (Olivoto and Nardino, 2021). The broad sense heritability ranged from 54.3% for fruit weight to 88.05 for vulnerability index. The studied traits were reported in a single factor (FA1) with most yield-related traits. Fruit yield and number of fruits had positive loadings in FA1, whereas vulnerability index, days to first female flower, and fruit weight had negative loadings. Genotypes ranking based on MGIDI values are depicted in **Figure 8**. The selected genotypes based on selection pressure (~15%) were G-3(DSG-29), G-2(DSG-7), and G-6(DSGVRL-18). The selected genotypes resulted in desirable positive selection gains for average performance of yield-attributing traits, *viz*., fruit yield per plant, number of fruits per plant, and fruit weight. These genotypes also recorded desirable negative gains for vulnerability index and earliness (**Supplementary Table 2**).

### Biochemical characterization

**Figure 9:**
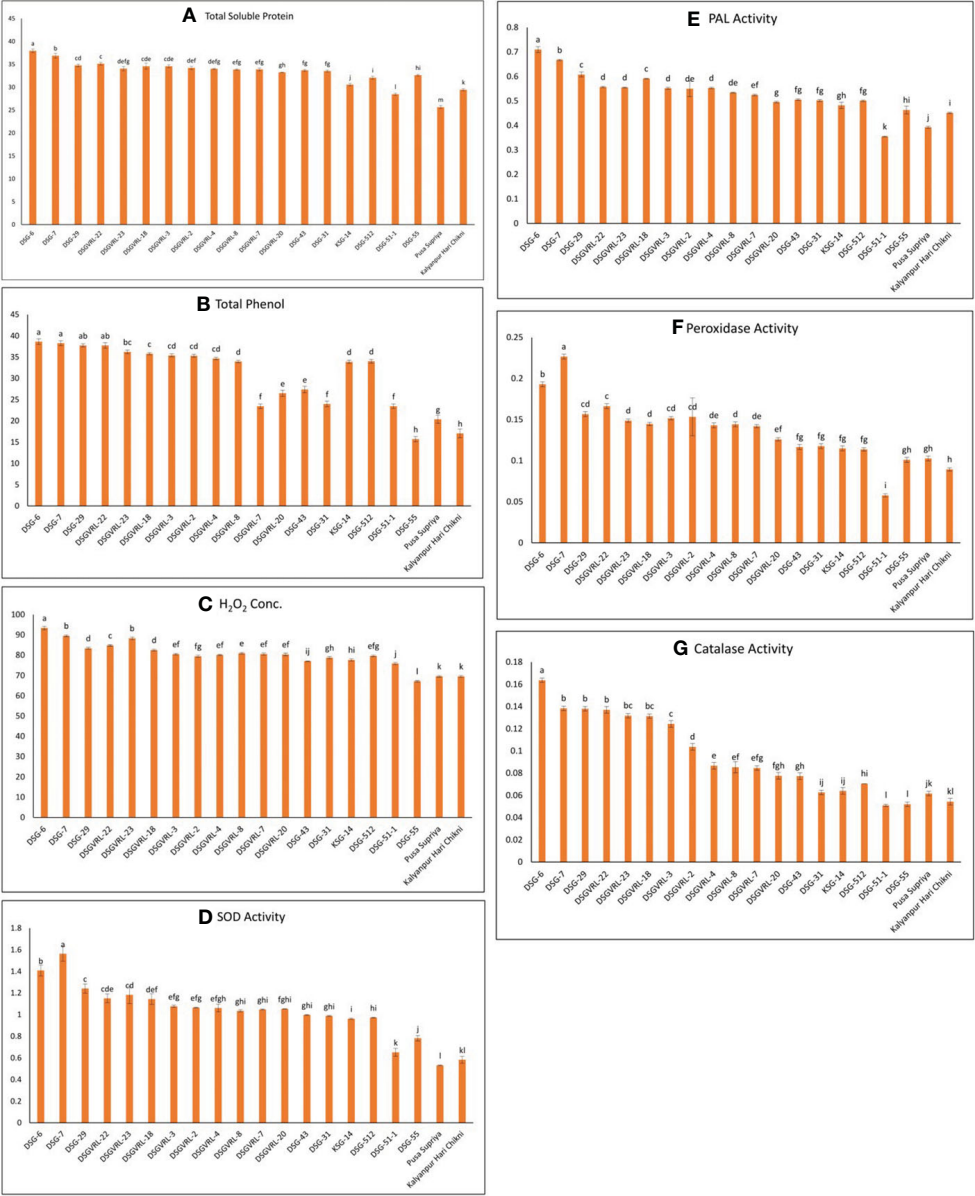
Graphical representation of total soluble protein content **(A)**, total phenol assay **(B)**, hydrogen peroxide assay **(C)**, superoxidase dismutase (SOD) **(D)**, phenyl ammonia lyase (PAL) **(E)**, peroxidase **(F)**, and catalase **(G)** in 20 genotypes of sponge gourd belongs to the different category of resistance and susceptible against ToLCNDV.

For biochemical characterization, eight resistant genotypes, four genotypes each of moderately resistant, moderately susceptible, and susceptible category, were selected based on their response against ToLCNDV disease. The resistant genotypes exhibited the highest level of all biochemical parameters compared with susceptible genotypes (**Figure 9**). The highest soluble protein was observed in resistant genotype DSG-6 (37.95 mg/g fresh weight), followed by DSG-7 (36.89 mg/g fresh weight) and lowest in susceptible genotype Pusa Supriya (25.65 mg/g fresh weight). The value of soluble protein of resistant genotypes was significantly higher than that of susceptible genotypes, whereas most of the moderately resistant and moderately susceptible genotypes are non-significant with each other. For total phenol content, resistant genotype DSG-6 recorded the highest value at 38.64 mg/g fresh weight, which is statistically at par with other resistant genotypes, *viz*., DSG-7, DSG-29, and DSGVRL-22. The lowest phenol content was observed in susceptible genotypes DSG-55, exhibiting a value statistically equivalent to that of Kalyanpur Hari Chikni. Resistant genotype DSG-6 recorded the highest H_2_O_2_ concentration, followed by DSG-7, statistically comparable with DSGVRL-23. In contrast, susceptible genotype Kalyanpur Hari Chikni exhibited the lowest concentration, statistically equivalent to Pusa Suripya. The highest SOD enzyme activity was observed in resistant genotype DSG-7, followed by DSG-6, whereas the lowest was in susceptible genotype Pusa Supriya. The accumulation of PAL enzyme activity was highest in resistant genotype DSG-6, followed by DSG-7 and DSG-29. However, the lowest PAL enzyme activity was recorded in susceptible genotypes DSG-51-1, followed by Pusa Supriya. The resistant genotype DSG-7 showed maximum peroxidase enzyme activity followed by DSG-6, whereas the lowest was recorded in susceptible genotype DSG-51-1. For catalase enzyme activity, resistant genotype DSG-6 recorded the highest value, followed by DSG-7, which is statistically at par with DSG-29 and DSGVRL-22. The lowest value of catalase enzyme activity was observed in susceptible genotype DSG-51-1, which is statistically equivalent to DSG-55.

## Discussion

ToLCNDV has become a significant threat to the cultivation of various crops across diverse plant families owing to its broad host range. In cucurbits, globally, it has caused substantial economic losses, and under epidemic conditions, it can result in total crop loss (100%) in sponge gourd (Islam et al., 2010, 2011; Kumar et al., 2019; Dhillon et al., 2020). To overcome this problem, host plant resistance against ToLCNDV incidence is the most effective and sustainable approach one should adapt for reducing the yield loss in sponge gourd. At present, most of the commercially grown sponge gourd varieties are susceptible to ToLCNDV. Therefore, extensive screening of the vast array of germplasm resources is imperative to identify the resistant sources, which can be used in resistant breeding programs of sponge gourd. However, screening the genotypes for resistance is cumbersome and challenging due to the unpredictable nature of the environmental conditions and vector populations under field conditions. Furthermore, screening of diverse genotypes in different environments in order to identify the most resistant genotype to *ToLCNDV* is an expensive venture. Thus, identifying an ideal test environment with excellent discrimination power for genotypes is of utmost importance. To deal with these problems, GGE biplot analysis is a simple and powerful tool for analyzing the genotypes using the environmental data. Hence, it helps in efficient assessment of genotypes and identification of best test location. Considering these facts, 50 diverse genotypes of sponge gourd were screened against ToLCNDV under natural conditions for 3 years. Furthermore, a subset of 20 diverse genotypes being identified based on the results of the preliminary screening were evaluated across four diverse environments during 2021.

### Identification of stable resistant source against ToLCNDV

In our study, eight genotypes showed stable resistance against ToLCNDV along with other desirable traits, *viz*., earliness, fruit number, and fruit yield, based on results of the preliminary screening for 3 years. These results were further validated through the mean vs. stability graph view of GGE biplot. The correlation study reveals that ToLCNDV infection severely affected the yield potential and other important traits, such as number of fruits, days to flowering, and fruit weight in sponge gourd. The cluster heat map categorized the resistant genotypes into two distinct groups, with five genotypes in group I and the remaining three in group III. Most of the susceptible commercial cultivars were clustered in group VII. Furthermore, the activity and accumulation of defense-related enzymes under disease conditions in sponge gourd genotypes with varied resistance level to ToLCNDV were also investigated. The total soluble protein, total phenol content, H_2_O_2_ concentration, SOD, PAL, peroxidase, and CAT activity were higher in resistant genotypes as compared with susceptible genotypes. Therefore, reduced activity of these enzymes can effectively aid in differentiating between resistant and susceptible genotypes in sponge gourd against ToLCNDV. These findings are in alignment with the previous reports in *Cucurbita pepo* infected with *zucchini yellow mosaic virus* (ZYMV) (Radwan et al., 2007) and tomato and bell pepper infected with *tomato mosaic virus* (Madhusudhan et al., 2009). Similar results were also obtained by Dieng et al., 2011; Sahu et al., 2012 and Sharma et al., 2021 in tomato infected by *tomato yellow leaf curl virus*.

In our study, genotypes exhibited significant genotype–environment interaction in response to ToLCNDV and other yield-related traits under multi-environmental trials. The combined ANOVA displayed significant variation due to genotype, environment, and genotype × environment interaction for VI, earliness, and other yield-related traits under the ToLCNDV disease condition. It was also noted that there was inconsistent performance of genotypes across the locations is attributed to the diverse environmental condition for disease incidence. Therefore, the presence of significant GEI reveals the importance of assessing the genotypes across multiple locations before ranking them against ToLCNDV resistance in sponge gourd. The host–pathogen interaction is very complex in nature and is influenced by many factors such as host genotype, pathogen, and environment. Hence, plant breeders must identify durable resistant genotypes with minimum environmental interaction across the environments. The GGE biplot analysis helps in simplification of complex GEI into different principal components and graphically represents them against various principal components (Yan and Tinker, 2006). In our study, first two principal component accounted for more than 90% of the total variance, affirming the comprehensive representation of total variability for VI and yield-related traits under ToLCNDV incidence. The mean vs. stability view of the GGE biplot reveals that DSG-6(G-1), DSG-7(G-2), DSG-29(G-3), DSGVRL-22(G-4), DSGVRL-23(G-5), DSGVRL-18(G-6), and DSGVRL-3(G-7), and DSGVRL-2(G-8) were positioned downstream of the AEA abscissa, thus indicating lower values of the vulnerability index and thereby considered as resistant. Although G-8 was more stable among these genotypes, which is revealed by its projection on AEA abscissa; however, it is not considered as an ideal genotype. Because stability is only meaningful when it is associated with higher performance of the respective trait (Yan and Tinker, 2006). Thus, for the vulnerability index, genotype DSG-7(G-2) was considered as the most ideal with higher disease resistance along with stable performance across the environments. DSG-29(G-3), DSG-7(G-2), DSG-6(G-1), and DSGVRL-18(G-6) were identified as the most desirable genotypes with stable resistance against ToLCNDV and also exhibiting higher performance in response to earliness, fruit weight, number or fruits, and fruit yield. Thus, genotypes identified as ideal and desirable with durable resistance, would be valuable genetic resources in future resistant breeding programs in sponge gourd against ToLCNDV. Similarly resistant genotypes were also identified in various crops against different pathogens (Parihar et al., 2017; Das et al., 2020; Singh et al., 2020; Sankar et al., 2021).

### Identification of genotypes with high yield potential and enhanced resistance

The ability of a genotype to combine higher yield performance with other important traits such as resistance should be utilized to assess their superiority instead of individual trait performance. Farmers’ acceptance of ToLCNDV resistant with low-yielding sponge gourd varieties is doubtful. To gain widespread and consistent acceptance from the growers, new cultivars must not only give high ToLCNDV resistance but also maintain or exceed the current yield level. This perspective highlights the yield traits’ importance and should be considered as primary breeding objective in improvement programs of sponge gourd. The other important traits like disease resistance only hold economic value when it is associated with an adequate yield level. To address this problem, the GYT biplot is used to evaluate genotypes for multiple traits. The GYT biplot ranks the genotypes’ superiority based on their ability to combine yield with various targeted traits and simultaneously highlighting their strength and weaknesses. It also fulfils all the conditions required for ATC graph, thus making it a valuable tool for genotype ranking. However, despite the GT biplot’s ability to illustrate the trait correlation and genotypes’ strength and weaknesses, it falls short in effective decision-making on acceptance and rejection of genotypes. Furthermore, the GT biplot does not meet the condition of no strong negative correlation among traits, thus making the ATC view ineffective (Yan and Frégeau-Reid, 2018). Traits like earliness, number of fruits, fruit yield, and average fruit weight are directly affected by ToLCNDV incidence, as revealed by the correlation graph and GT biplot. Thus, the GYT biplot based on these traits helps in identification of genotypes DSG-29 (G-3), followed by DSG-7 (G-2) and DSGVRL-18 (G-6) as the most stable and ideal to combine a high level of yield along with other important traits like ToLCNDV resistance, earliness, and more number of fruits per plant under disease incidence. MGIDI is an innovative approach, which has proven to be highly effective in numerous breeding programs, paving the way for more efficient and successful selection strategies (Nelimor et al., 2020; Pour-Aboughadareh et al., 2021; Nardino et al., 2022; Yue et al., 2022a, b; Dhand and Garg, 2023; Memon et al., 2023; Singamsetti et al., 2023). According to MGIDI index values, genotypes DSG-29 (G-3), DSG-7 (G-2), and DSGVRL-18 (G-6) have higher mean performance and desirable selection gains for yield-attributing traits and resistance to *ToLCNDV*. Apart from the selected genotypes, DSGVRL-23 (G-5) and DSGVRL-22 (G-4) were placed at the cutting points and could be exploited for resistance and yield-related traits in sponge gourd.

### Identification of ideal environment for screening

The application of the GGE biplot in identification of ideal testing location facilitates in minimizing the trial costs and optimal resource allocation without comprising the trial heritability and genetic gain under selection (Yan, 2001; Yan and Tinker, 2006; Yan and Holland, 2010). Representativeness is the most significant factor in deciding the suitability of the test environment for genotype evaluation, provided it possesses a sufficient discrimination ability (Yan et al., 2007). The environment vector length is used to measure the discrimination ability, and the smaller degree of angle with the AEA abscissa indicates representativeness of these test environments (Yan and Tinker, 2006). In our study, E1 and E2 have more discriminative power, whereas E4 is the least discriminating for disease screening and fruit yield under ToLCNDV disease incidence. Thus, test environments E1 and E2 with high discrimination and representativeness were recognized as the ideal testing locations for evaluation of advanced breeding lines of sponge gourd against ToLCNDV resistance. These results are further confirmed by the first report of ToLCNDV infestation in *Luffa* at ICAR-IARI, New Delhi, by Sohrab et al. (2003) and the findings of Islam et al. (2010, 2011). However, with respect to E4, it was not a desirable environment for screening due to lower incidence of disease during the spring–summer season, which may be due to reduced proliferation of white flies.

The “which-where-won” view revealed the existence of distinct mega-environments among the tested environments. This environment grouping indicates the presence of genotype × environment interaction, suggesting that the genotypes perform differently across environments. Moreover, these mega-environments can be used to identify genotypes that are specifically adapted to specific locations (Yan, 2011). In the present investigation, all the tested environments were separated into two mega environments. This may be due to divergence in the ToLCNDV severity due to genotypic and environmental variations. The environment, *kharif* season, which is the most desirable, was separated from the spring season, thus indicating that the environmental conditions during the *kharif* season are more conducive for ToLCNDV incidence. A strong positive correlation and similarity in the genotype performance was also observed within the tested environment of a particular mega environment. The resistant genotypes did not align with any specific sectors forming the mega-environments, indicating their consistent disease reaction across all tested environments. Furthermore, vertex genotypes with no environment in its sector indicate poor performance for that particular trait across the environment. Therefore, in our study, poor performance of resistant genotypes for VI indicates their resistant nature across all the environments. The genotypes DSG-29(G-3), followed by DSG-7(G-2) and DSGVRL-18(G-6), were observed as the winning genotypes concerning resistance against ToLCNDV, earliness, and yield-attributing traits under disease conditions in sponge gourd. The identified environmental grouping provides a valuable framework for sponge gourd resistance breeding against ToLCNDV, which should be further validated through multi-location testing over several years, as emphasized from previous studies of Yan et al. (2000); Silva et al. (2011), Phuke et al. (2017), Sánchez-Martín et al. (2017); Das et al. (2020), and Sankar et al. (2021). The performance and stability demonstrated by the selected material needs to be further tested in the eastern and southern parts of India for future advancement of elite cultivars resistant to ToLCNDV in sponge gourd.

## Conclusion

These results revealed the significance of multi-environment testing for evaluation of genotypes against ToLCNDV resistance. The tested environments were categorized into two distinct mega-environments for evaluation under ToLCNDV incidence with different winning genotypes, which confirms the GEI. Based on GGE biplot results, Based on GGE biplot results, mega-environment with *kharif* season was identified as the most ideal test environment for natural screening due to its discrimination and representative power. Among tested genotypes, DSG-29 (G-3) was identified as the ideal genotype and DSG-7 (G-2), DSGVRL-18 (G-6), and DSG-6 (G-1) as the desirable genotypes exhibiting higher resistance levels along with higher yield, earliness, and other yield-attributing traits. The hurdle of late flowering associated with ToLCNDV infestation can be overcome by utilizing early-maturing genotypes such as DSG-29(G-3), DSG-7(G-2), DSGVRL-23(G-5), and DSGVRL-18(G-6) identified in the study. Thus, the genotypes identified can be utilized for sponge gourd improvement programs to develop cultivars resistant to ToLCNDV with enhanced productivity and stability.

## Data availability statement

The original contributions presented in the study are included in the article/**Supplementary Material**. Further inquiries can be directed to the corresponding authors.

## Author contributions

JS: Conceptualization, Data curation, Formal analysis, Funding acquisition, Investigation, Methodology, Project administration, Resources, Supervision, Validation, Visualization, Writing – original draft, Writing – review & editing. AM: Conceptualization, Investigation, Methodology, Project administration, Resources, Supervision, Writing – review & editing. DS: Data curation, Formal Analysis, Methodology, Software, Supervision, Validation, Visualization, Writing – review & editing. BM: Data curation, Formal analysis, Investigation, Methodology, Supervision, Validation, Writing – original draft. AS: Data curation, Funding acquisition, Investigation, Methodology, Resources, Supervision, Visualization, Writing – original draft, Writing – review & editing. AN: Data curation, Investigation, Methodology, Supervision, Writing – original draft, Writing – review & editing. YL: Formal analysis, Methodology, Resources, Software, Validation, Writing – original draft, Writing – review & editing. BT: Funding acquisition, Project administration, Resources, Supervision, Validation, Visualization, Writing – review & editing. SD: Funding acquisition, Investigation, Methodology, Project administration, Resources, Supervision, Validation, Writing – review & editing. JR: Formal analysis, Funding acquisition, Methodology, Project administration, Resources, Supervision, Validation, Visualization, Writing – review & editing. NS: Formal analysis, Methodology, Software, Supervision, Validation, Writing – review & editing. NK: Data curation, Investigation, Methodology, Validation, Writing – review & editing. KM: Methodology, Software, Validation, Writing – review & editing.
