# Supplementary material for: Identification of new stable resistant sources and assessing agro-morphological performance of sponge gourd germplasm against Tomato Leaf curl New Delhi Virus incidence
Source: Front Plant Sci. 2024 Apr 24;15:1373352. doi: 10.3389/fpls.2024.1373352 (PMC11076759; doi:10.3389/fpls.2024.1373352)
Supplement: Supplementary file 1 [file DataSheet_1.docx]

Supplementary Table 1: Genotypes of sponge gourd used in the present study, their sources and vulnerability index against *ToLCNDV* based on three year screening during 2018, 2019, and 2020.

| S.no | Genotypes | Genotype Code | Source | Fruit colour | Resistance category | Mean Vulnerability index of 2018, 2019, and 2020 |
| --- | --- | --- | --- | --- | --- | --- |
| 1 | DSG-6 | G-1 | IARI, New Delhi | Dark Green | R | 4.44 |
| 2 | DSG-7 | G-2 | IARI, New Delhi | Light green | R | 3.23 |
| 3 | DSG-29 | G-3 | IARI, New Delhi | Dark Green | R | 8.00 |
| 4 | DSGVRL-22 | G-4 | IARI, New Delhi | Green | R | 11.19 |
| 5 | DSGVRL-23 | G-5 | IARI, New Delhi | Dark green | R | 15.13 |
| 6 | DSGVRL-18 | G-6 | IARI, New Delhi | Dark Green | R | 19.24 |
| 7 | DSGVRL-3 | G-7 | IARI, New Delhi | Dark Green | R | 21.85 |
| 8 | DSGVRL-2 | G-8 | IARI, New Delhi | Dark Green | R | 24.27 |
| 9 | DSGVRL-4 | G-9 | IARI, New Delhi | Dark Green | MR | 26.76 |
| 10 | DSGVRL-8 | G-10 | IARI, New Delhi | Dark Green | MR | 28.35 |
| 11 | DSGVRL-7 | G-11 | IARI, New Delhi | Dark Green | MR | 31.08 |
| 12 | DSGVRL-20 | G-12 | IARI, New Delhi | Light green | MR | 32.58 |
| 13 | DSGVRL-21 | G-13 | IARI, New Delhi | Light green | MR | 33.13 |
| 14 | DSGVRL-1 | G-14 | IARI, New Delhi | Light green | MR | 35.14 |
| 15 | DSGVRL-15 | G-15 | IARI, New Delhi | Green | MR | 40.36 |
| 16 | DSGVRL-6 | G-16 | IARI, New Delhi | Dark Green | MR | 44.41 |
| 17 | DSGVRL-13 | G-17 | IARI, New Delhi | Light green | MR | 45.58 |
| 18 | DSGVRL-12 | G-18 | IARI, New Delhi | Light green | MR | 49.30 |
| 19 | DSGVRL-14 | G-19 | IARI, New Delhi | Light green | MR | 49.98 |
| 20 | DSG-43 | G-20 | IARI, New Delhi | Dark green | MS | 53.19 |
| 21 | NSG-28 | G-21 | Nirmal Seeds, Pantnagar | Dark green | MS | 56.07 |
| 22 | DSG-31 | G-22 | IARI, New Delhi | Light green | MS | 56.59 |
| 23 | DSGVRL-16 | G-23 | IARI, New Delhi | Light green | MS | 61.47 |
| 24 | DSGVRL-17 | G-24 | IARI, New Delhi | Light green | MS | 60.51 |
| 25 | DSGVRL-24 | G-25 | IARI, New Delhi | Dark green | MS | 65.64 |
| 26 | DSGVRL-25 | G-26 | IARI, New Delhi | Light green | MS | 67.62 |
| 27 | DSGVRL-5 | G-27 | IARI, New Delhi | Light green | MS | 70.41 |
| 28 | DSGVRL-19 | G-28 | IARI, New Delhi | Light green | MS | 70.46 |
| 29 | KSG-14 | G-29 | Kalyanpur, UP | Dark green | MS | 71.70 |
| 30 | DSG-512 | G-30 | IARI, New Delhi | Dark green | MS | 72.74 |
| 31 | DSGVRL-9 | G-31 | IARI, New Delhi | Light green | MS | 72.02 |
| 32 | DSGVRL-10 | G-32 | IARI, New Delhi | Light green | MS | 74.69 |
| 33 | DSGVRL-11 | G-33 | IARI, New Delhi | Light green | MS | 75.37 |
| 34 | DSG-95 | G-34 | IARI, New Delhi | Dark green | S | 76.50 |
| 35 | Pusa Sneha | G-35 | IARI, New Delhi | Dark green | S | 76.84 |
| 36 | DSG-507 | G-36 | IARI, New Delhi | Dark green | S | 77.88 |
| 37 | DSG-508 | G-37 | IARI, New Delhi | Light green | S | 79.21 |
| 38 | DSG-509 | G-38 | IARI, New Delhi | Dark green | S | 81.81 |
| 39 | CHSG-1 | G-39 | HARP, Ranchi | Green | S | 82.14 |
| 40 | CHSG-2 | G-40 | HARP, Ranchi | Green | S | 82.27 |
| 41 | DSG-506 | G-41 | IARI, New Delhi | Green | S | 83.35 |
| 42 | JSLG-55 | G-42 | Junagad, Gujarat | Light green | S | 84.57 |
| 43 | DSG-47 | G-43 | IARI, New Delhi | Dark green | S | 86.12 |
| 44 | DSG-510 | G-44 | IARI, New Delhi | Dark green | S | 87.60 |
| 45 | DSG-511 | G-45 | IARI, New Delhi | Dark green | S | 89.78 |
| 46 | PSG-9 | G-46 | PAU, Ludhiana | Green | S | 91.16 |
| 47 | DSG-51-1 | G-47 | IARI, New Delhi | Light green | S | 93.48 |
| 48 | DSG-55 | G-48 | IARI, New Delhi | Light green | S | 95.06 |
| 49 | Pusa Supriya | G-49 | IARI, New Delhi | Light green | S | 95.51 |
| 50 | Kalyanpur Hari Chikni | G-50 | IARI, New Delhi | Dark green | S | 96.10 |

Supplementary Table 2: Selection gains for mean performance of twenty sponge gourd genotypes evaluated under *ToLCNDV* conditions based on MGIDI values.

| S.no | Trait | Factor | sense | Goal | X_o_ | X_s_ | SD | SD% | h^2^ | SG | SG% |
| --- | --- | --- | --- | --- | --- | --- | --- | --- | --- | --- | --- |
| 1 | VI | FA1 | decrease | Yes | 39.7 | 7.87 | -31.8 | -80.2 | 0.88 | -28 | -70.6 |
| 2 | DFFFA | FA1 | decrease | Yes | 46.4 | 40.8 | -5.61 | -12.1 | 0.731 | -4.1 | -8.84 |
| 3 | AFW | FA1 | increase | Yes | 121 | 117 | -3.94 | -3.26 | 0.543 | -2.14 | -1.77 |
| 4 | FPP | FA1 | increase | Yes | 9.93 | 17.6 | 7.69 | 77.4 | 0.793 | 6.09 | 61.3 |
| 5 | FYLP | FA1 | increase | Yes | 1.19 | 2.03 | 0.839 | 70.4 | 0.798 | 0.669 | 56.1 |

VI- Vulnerability index, DFFFA- Days to first female flower anthesis, AFW- Average fruit weight (g), FPP- Number of Fruits per plant, FYLP- fruit yield per plant (kg/plant); FA1: factor 1; SD, selection differential; SD%, percent selection differential; SG, selection gain; SD%, percent selection gain ;Xo, mean of the original population; Xs, mean of the selected hybrids.

Supplementary Figure 1: Mean *vs*. Stability view of the GGE biplot of 50 sponge gourd genotypes across 3 testing environments for vulnerability index (A), fruit yield per plant (B), number of fruits per plant (C), days to first male flower anthesis (D), days to first female flower anthesis (E), days to first fruit harvest (F), fruit diameter (G), fruit length (H), and average fruit weight (E). Scaling = 0, centring = 2, SVP = 1. Numbers correspond to genotypes as listed in supplementary Table 1.


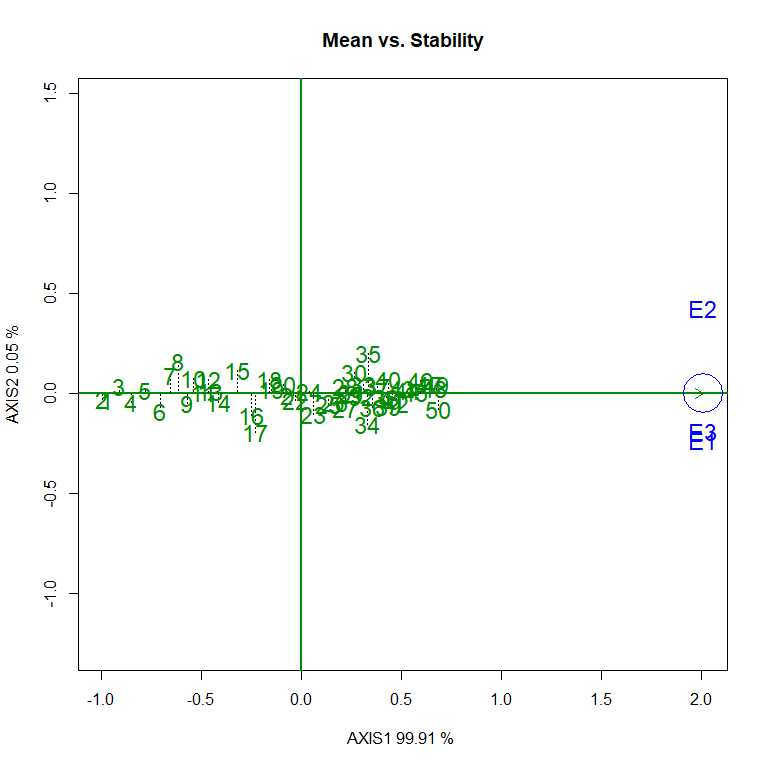

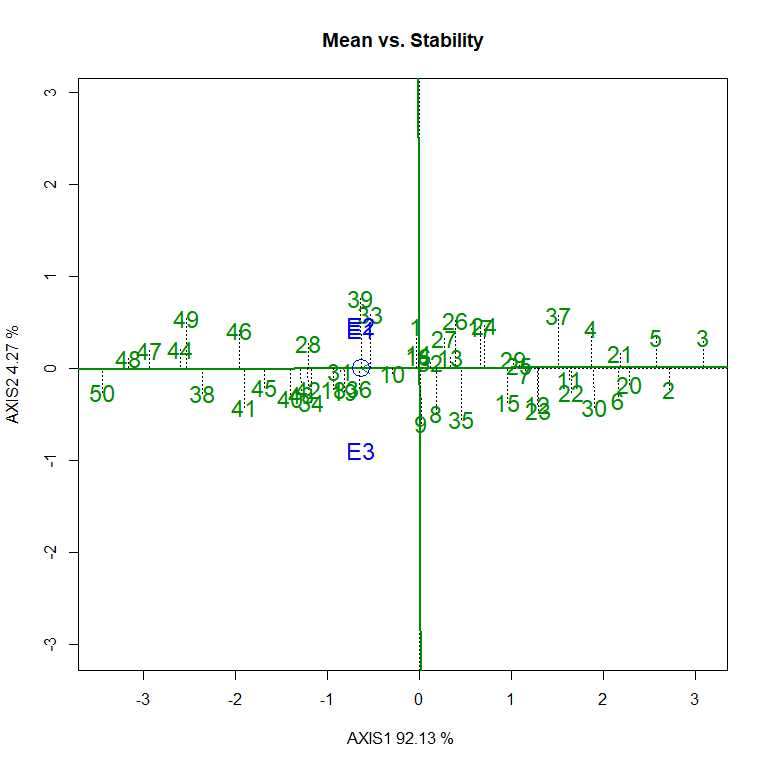

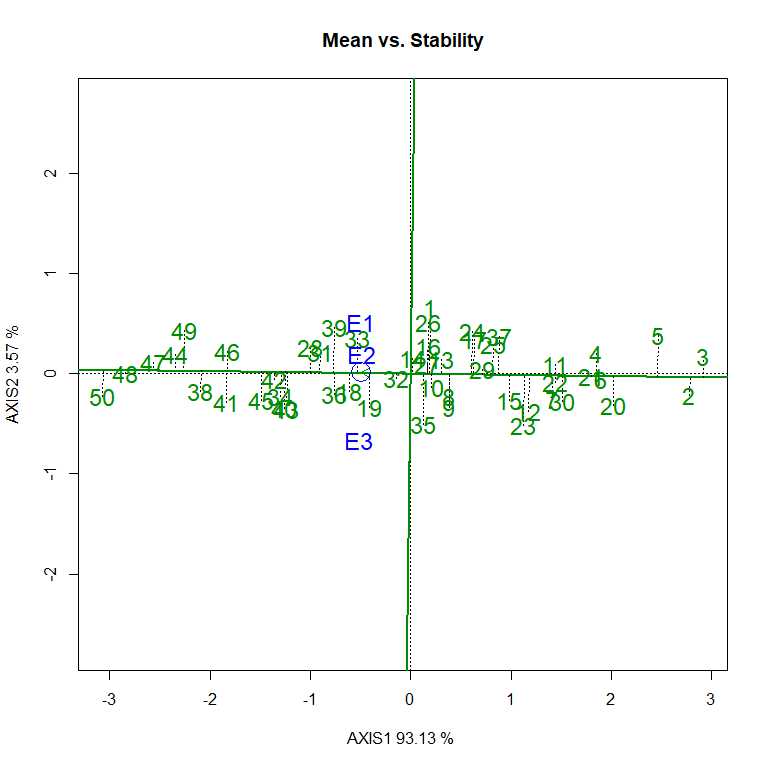

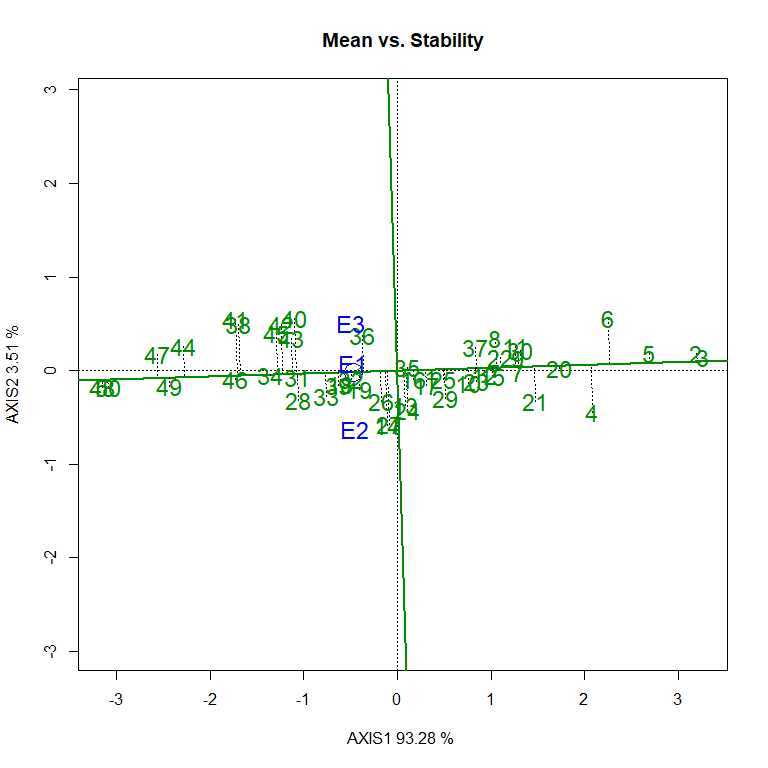

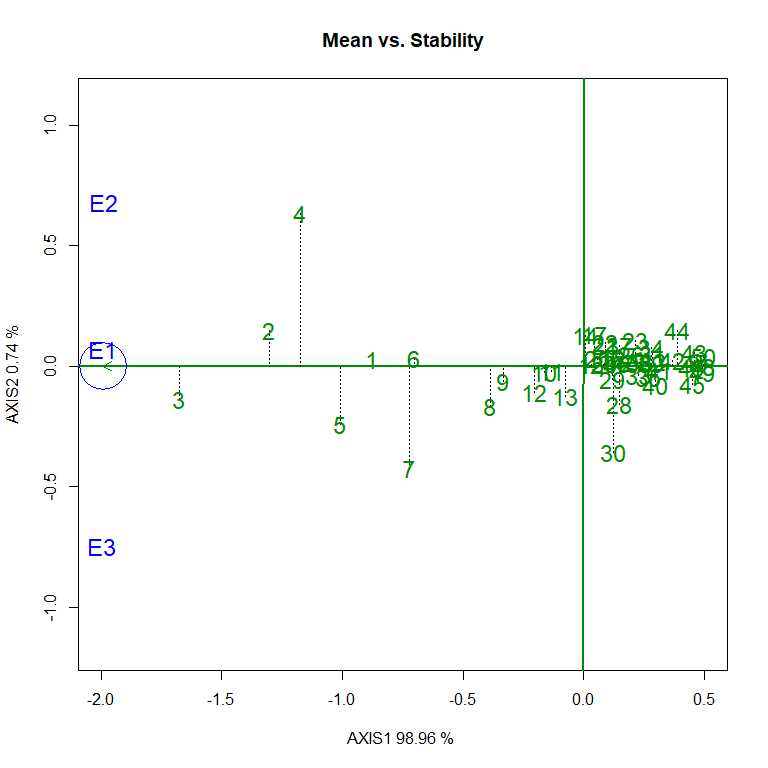

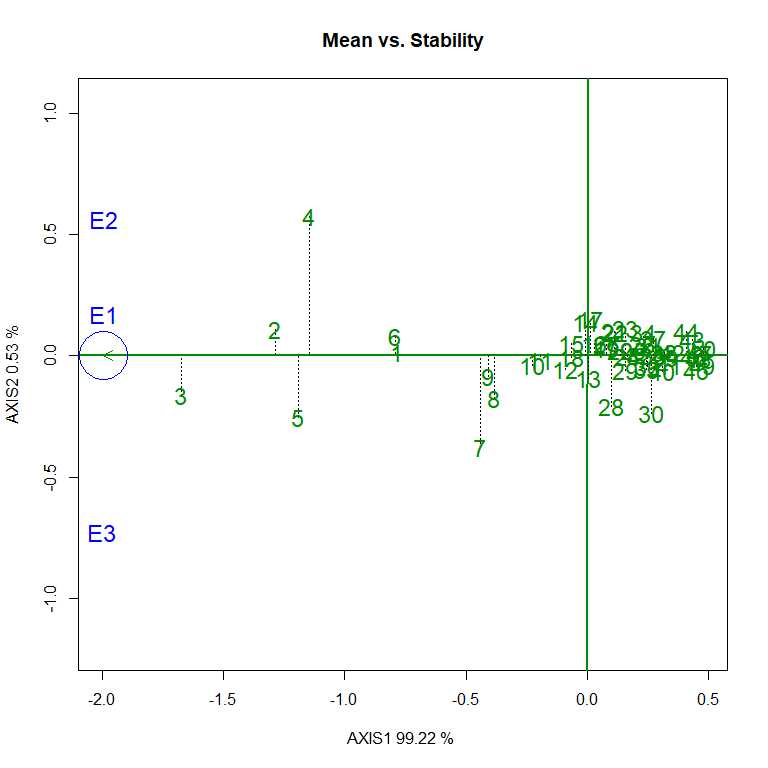

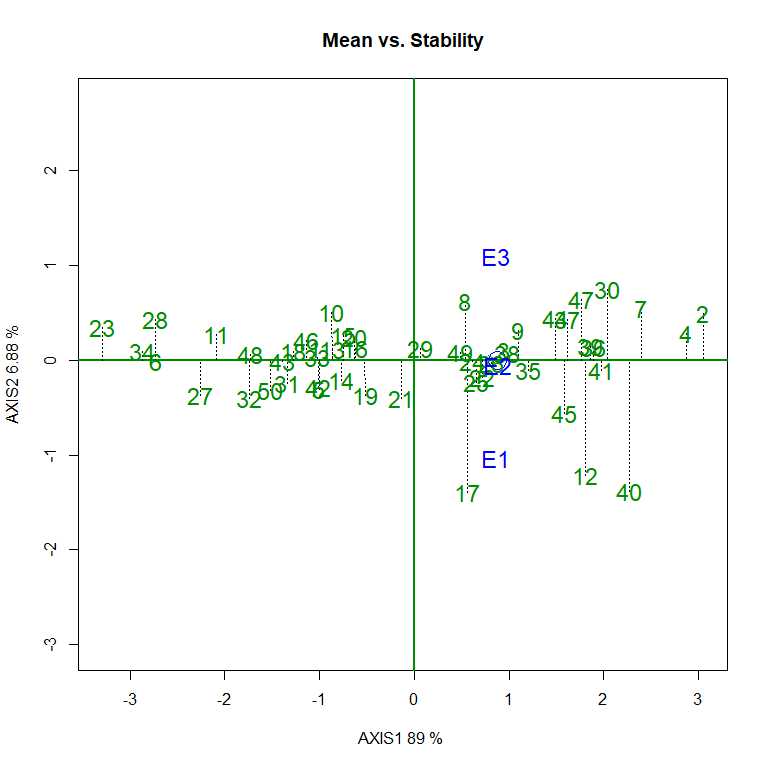

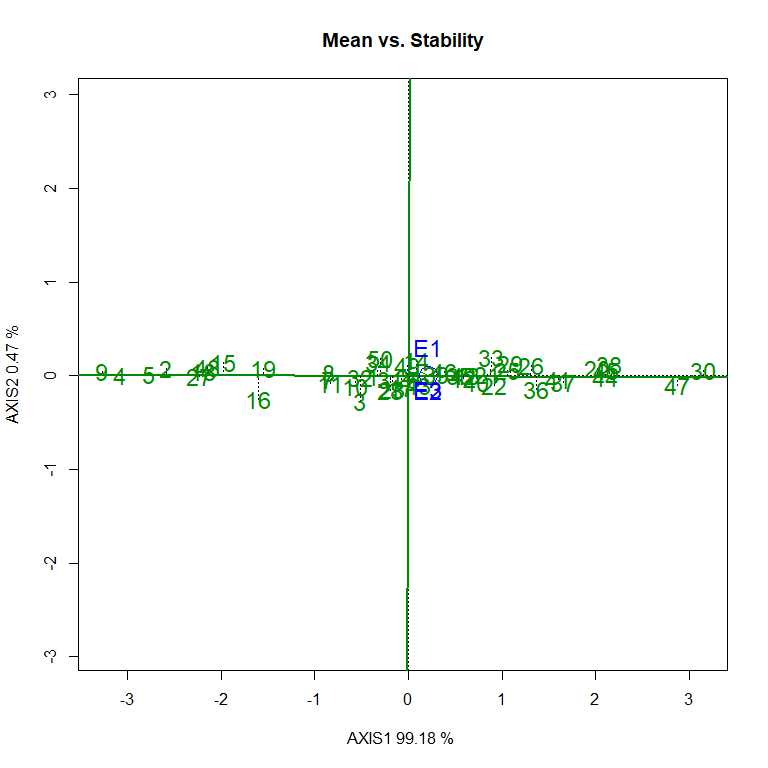

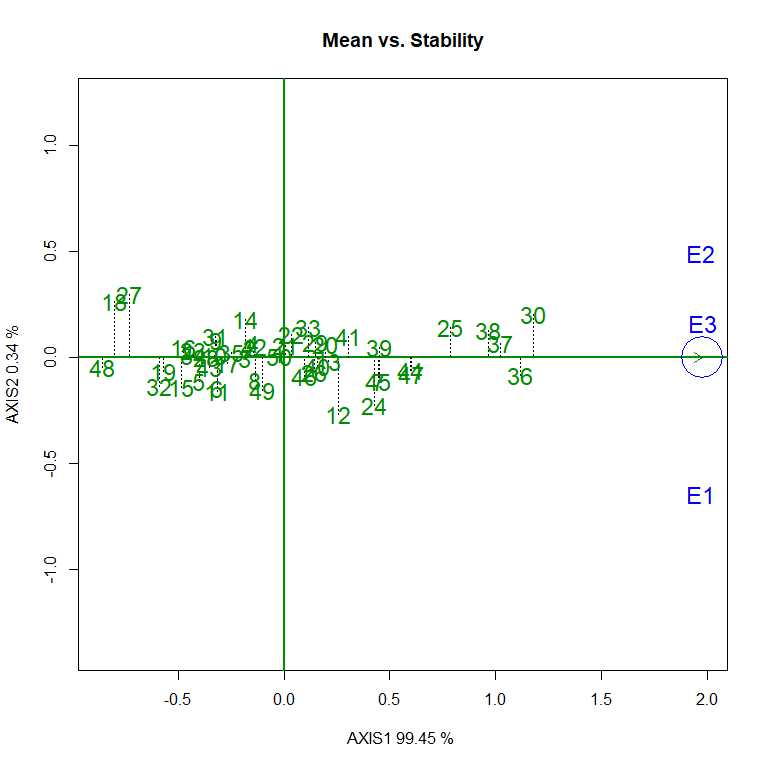


**A**

**B**

**C**

**D**

**E**

**F**

**G**

**I**

**H**
